# An annotated checklist of the leaf beetles (Coleoptera, Chrysomelidae) from El Salvador, with additions from the Bechyné collection in the Royal Belgian Institute of Natural Sciences

**DOI:** 10.3897/zookeys.856.32017

**Published:** 2019-06-17

**Authors:** Martijn Van Roie, Frederik De Wint, Ayse Güngor, Charlotte Huyghe, Wouter Dekoninck, Lukáš Sekerka

**Affiliations:** 1 Biodiversity Inventory for Conservation (BINCO) vzw, Walmersumstraat 44, 3380 Glabbeek, Belgium Biodiversity Inventory for Conservation Brecht Belgium; 2 Department of Biology, Ecosystem Management Research Group, University of Antwerp, Universiteitsplein 1, 2610 Wilrijk, Belgium University of Antwerp Antwerp Belgium; 3 Royal Belgian Institute for Natural Sciences, Vautierstraat 29, 1000 Brussels, Belgium Royal Belgian Institute for Natural Sciences Brussels Belgium; 4 Department of Entomology, National Museum, Cirkusová 1740, CZ-193 00, Praha 9 – Horní Počernice, Czech Republic Department of Entomology, National Museum Prague Czech Republic

**Keywords:** Digitization, Central America, Neotropics, Museum

## Abstract

A checklist of the species of leaf beetles (Coleoptera: Chrysomelidae) of El Salvador is presented based on data from literature and a digitization project of the Bechyné collection of the Royal Belgian Institute of Natural Sciences (RBINS). The RBINS collections contain a total of 2797 individual chrysomelid specimens from El Salvador, sorted into 89 species and 132 genera. In total, the current checklist contains 420 species, of which 33 are new records for El Slavador from the Bechyné collection. In these collections, there are also ten nomina nuda named by Bechyné, which need further study. The leaf beetle diversity in El Salvador, partly due to the country’s unstable political history, remains poorly studied, and many (new) species await discovery. This checklist provides a baseline for further study in El Salvador and nearby region.

## Introduction

The description and inventory of biodiversity is facing hard times due to budgetary problems and the decline of taxonomists (Drew 2011). This is also certainly the case for leaf beetles (Coleoptera: Chrysomelidae). Nonetheless, leaf beetles can be of economic significance (Livia 2006), often possess interesting life histories (e.g., the semi-aquatic lifestyle of most Donaciinae, Kleinschmidt and Kölsch (2011)), and can display complex behavior like eusociality (Windsor et al. 2013). Leaf beetles are a taxonomically complex group and, although some regions where the diversity of this group is relatively well known (like the Palearctic and Nearctic regions), other areas like the Neotropics are in desperate need of more study.

One of the most important early studies on Neotropical Chrysomelidae was done for the multipartite series of the Biologia Centrali Americana (Baly and Champion 1885–1894; Jacoby 1880–1892; 1888–1892). The series remains the most complete source of information today on insect fauna of Central America. This work collated information from previous taxonomists, e.g., Crotch, Illiger, Harold and Baly, who published many monographs and species descriptions within Alticini, Eumolpinae, Cryptocephalinae and other subfamilies. In approximately the same period (1850–1862), Boheman had published his ‘Monographia Cassididarum’ dealing with the subfamily Cassidinae (Boheman 1850–1862). These early works were followed by the ‘Coleopterorum Catalogus’, published in multiple volumes, which represented the first actual checklist (Clavareau 1913a, 1913b, 1914; Heikertinger and Csiki 1939, 1940; Pic 1913; Spaeth 1914; Weise 1911, 1916, 1924). Further checklists include Blackwelder (1946), but, as stated by Furth and Savini (1996), this list contained mainly information derived from the ‘Coleopterorum Catalogus’. After the 1940’s, the works on Neotropical Chrysomelidae done by Jan Bechyné and his wife Bohumila Bechyné produced an impressive list of 188 publications about the Chrysomelidae from the Neotropical, Afrotropical and Palearctic regions, with a main focus on the subfamilies Eumolpinae and Galerucinae including Alticini (Seeno et al. 1976). Jan and Bohumila Bechyné described many species, and therefore significantly contributed to the knowledge of Neotropical Chrysomelidae. Another significant work is Scherer (1962) with an identification key to the genera of Neotropical Alticini (Scherer 1962). One of the most prolific taxonomists working on Neotropical leaf-beetles was Francisco Monrós (1922–1958). The Bechnynés’ works focused mainly on describing new taxa, and less on revising previous works and often ignored biological aspects of the taxa. Francisco Monrós focused his studies on detailed complex revisions of particular groups and include numerous ecological observations on host plants and behavior of the species. He published mainly on Criocerinae, Clytrini, Fulcidacini, and Lamprosomatinae. He was in the process of writing his opus magnum ‘Los géneros de Chrysomelidae’, but unfortunately due to his premature death only the first volume was published covering Sagrinae, Donaciinae, and Criocerinae (Monrós 1960).

Records from El Salvador are relatively rare (e.g., see Rodrigues and Mermudes 2016)). The country is undersampled for insects and this can be demonstrated by the fact that only two chrysomelid beetles are recorded from El Salvador in the ‘Biologia Centrali Americana’ series. The most intensive effort to construct a national list for the family dates back to 1960 with three main works by the Bechynés (Bechyné 1954; Bechyné and Bechyné 1960; 1963). Another list of Chrysomelidae from El Salvador is found in the two-part series ‘Lista de Insectos Clasificados de El Salvador’ (Berry and Salazar 1957, Berry 1959). However, these are inadequate because most Chrysomelidae species identifications include the statements “probably” and “proximately”, lacking information on who identified the species and depository of the specimens. Moreover, there are many misspellings and misinterpretations in species and author names and some species listed in Berry and Salazar (1957) are not included in Berry (1959) without any explanation. The occurrence data of these listed species should be verified by examination of material. Part of the material collected by Berry is deposited in the National Museum of Natural History (UNSM), Washington DC, USA. The Berry lists were unknown to most of subsequent authors, who explicitdly recorded species mentioned in the lists as new to El Salvador. This is particularly obvious in Cassidinae, which represent one of the best known chrysomelid subfamilies of the world.

Much of the Bechyné material from El Salvador was unfortunately lost during their trip from Europe to Brazil (Furth 2018, in litteris), but a total of 32 boxes remains in the Royal Belgian Institute of Natural Sciences (RBINS), and they contain hundreds of specimens of the Bechyné collection. These include numerous paratypes of the subfamily Eumolpinae and tribe Alticini.

We carried out a literature search, seeking any records of leaf beetles for El Salvador, with the main goal of constructing an updated and annotated checklist. Additionally, we added species data from the Bechyné El Salvador collection from the RBINS. The results of this work are presented here.

## Materials and methods

### Literature search

During our literature study, we accessed multiple historical references from eminent contributors to chrysomelid taxonomy, such as Bechyné, Stål, Jacoby, Baly, Blake, Harold, Illiger, and others as mentioned in the introduction. We also scouted some existing checklists from neighboring countries for references of El Salvadorean Chrysomelidae (e.g., Furth (2006), Furth (2013), Maes and Staines (1991)) as well as subfamily checklists for Central America level (e.g., Furth and Savini (1996)). A complete list of references containing records for El Salvador are indicated in our Results section. Species in Berry and Salazar (1957) and Berry (1959) which used the statements “probably” and “proximately” were not added in the present checklist to avoid incorrectly identified species.

### Specimen digitization

Eighteen full and 14 partially-filled insect drawers with material from El Salvador were digitized by volunteers (following the protocol described in Merckx et al. (2018)). For this, at least one specimen per species was photographed in dorsal, lateral and frontal views, using an Olympus TG4 digital camera with focus stacking functionality (see Mertens et al. (2017), where this method is discussed thoroughly). Pictures of these specimens were stacked using the Helicon Focus software (HeliconSoft Ltd, Kharkiv, Ukraine). A total of 42 specimens from El Salvador belonging to the genus *Calligrapha* Chevrolat that had already been digitized in a previous project were added to the database (Merckx et al. 2018). Furthermore, a high-resolution picture was taken of every insect drawer; such that all specimens have a clear dorsal picture, making intraspecific variation clear. The original labels of all specimens in the Bechyné El Salvador collection were also digitized, however for this manuscript the months of dates were changed to roman numerals to avoid confusion. The pictures and accompanying data are publicly available on-line at the RBINS online database (http://collections.naturalsciences.be/ssh-entomology).

### Taxonomy

We followed the system of Bouchard et al. (2011) for division of subfamilies and tribes. Names found in literature were screened for any nomenclatural changes in their relevant literature and/or were checked by experts on the respective groups (see acknowledgements). If a species record in literature had an outdated nomenclature, the name under which it was recorded is given under remarks at the relevant species checklist.

### Used abbreviations

We used the following abbreviations for institutes:

**RBINS** Royal Belgian Institute of Natural Sciences;

**BMNH** British Museum of Natural History;

**DBET** collection of Lech Borowiec, Wroclaw, Poland;

**LSC** collection of Lukáš Sekerka, Prague, Czech Republic;

**USNM**National Museum of Natural History, Smithsonian Institution, Washington DC, USA.

## Results

A total of 2.797 individual chrysomelid specimens from El Salvador, sorted into 89 species and 132 genera in the RBINS collections were digitized. A full list of the specimens included in the RBINS can be found below. Included in the collection were ten *nomina nuda*, namely: *Antitypona* sp. (Manuscript species – Eumolpinae), *Brachypnoea* sp. *1* (Manuscript species – Eumolpinae), *Brachypnoea* sp. *2* (Manuscript species – Eumolpinae), *Hylax* sp. (Manuscript species – Eumolpinae), *Percolaspis* sp. *1* (Manuscript species – Eumolpinae), *Percolaspis* sp. *2* (Manuscript species – Eumolpinae), *Phanaeta* sp. (Manuscript species – Eumolpinae), *Chaetocnema* sp. (Manuscript species – Galerucinae, Alticini), *Phyllotreta* sp. (Manuscript species – Galerucinae, Alticini) and *Walterianella* sp. (Manuscript species – Galerucinae, Alticini). We chose to not give the names and photographs of these undescribed species here to avoid the cluttering of invalid names in the literature.

**Figure 1.**
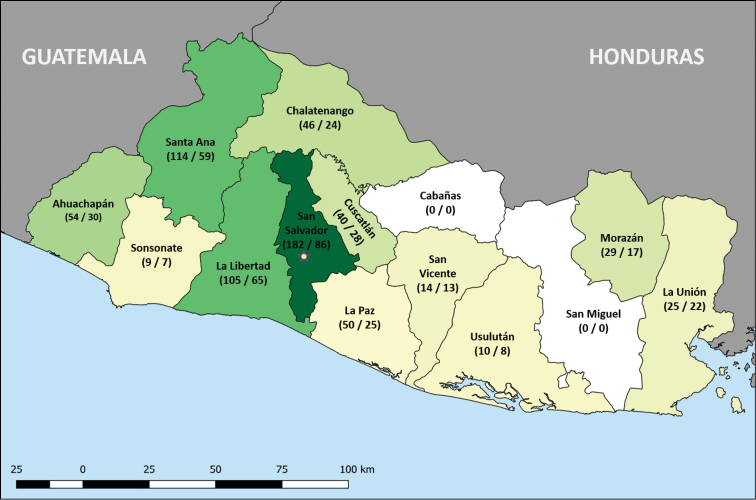
Heatmap of the species recorded per department of El Salvador. The darker the colour, the more species have been recorded. Numbers between brackets indicate number of species (first number) and number of genera (second number): (Species / Genera).

We noted a severe undersampling of Cryptocephalinae and Criocerinae. For example, Vencl et al. (2004) (Criocerinae) indicated the range of a species distribution (e.g., Mexico to Panama), but lacked specific records from El Salvador of which the authors are aware. Such unspecific records were left out of the present checklist. In total, the literature search led to a total of 385 chrysomelid species know to occur in El Salvador. Together with the Bechyné collection, this led to a combined total of 420 species (Figure 1 and Suppl. material 1: Table S1).

Suppl. material 1: Table S1 displays the number of species and genera per subfamily for each district. The departments with the highest species count to date are San Salvador (182 species), La Libertad (105 species), Santa Ana (114 species), Ahuachapán (54 species), La Paz (50 species) and Chalatenango (46 species). No records from both San Miguel and Cabañas could be found, nor were there any specimens from these departments included in the RBINS collections. Below is the checklist of species from El Salvador taken from literature (the reference indicated between brackets after every record) and from the Bechyné collection in the RBINS. Additionally, some extra records of Cassidinae were added from other sources known to one of the authors. If the authors were aware of any nomenclatural changes, this is indicated under “remarks”.

### Checklist of chrysomelid species from EL Salvador

#### Subfamily Bruchinae

**1. *Acanthoscelidesargillaceus* (Sharp, 1885)**

**Remarks.**Berry (1959) mentions *Acanthoscelidesarmitagei* Pic, 1931 as being present in El Salvador, but does not give specific records. This taxon was synonymized with *A.argillaceus* (Sharp, 1885) by Kingsolver (1969). Johnson (1990) also mentioned occurence in El Salvador without precise data.

**2. *Acanthoscelidesbrevipes* (Sharp, 1885)**

**Remarks.**Johnson (1990) mentions this species as being present in El Salvador, but does not give specific records.

**3. *Acanthoscelidesclitellarius* (Fahraeus, 1839)**

**Remarks.**Johnson (1990) mentions this species as being present in El Salvador, but does not give specific records.

**4. *Acanthoscelidesdesmoditus* Johnson, 1983**

**Remarks.**Johnson (1990) mentions this species as being present in El Salvador, but does not give specific records.

**5. *Acanthoscelidesdifficilis* (Sharp, 1885)**

**Remarks.**Johnson (1990) mentions this species as being present in El Salvador, but does not give specific records.

**6. *Acanthoscelidesgriseolus* (Fall, 1910)**

**Remarks.**Johnson (1990) mentions this species as being present in El Salvador, but does not give specific records.

**7. *Acanthoscelidesguazumae* Johnson & Kingsolver, 1971**

**Remarks.**Johnson (1990) mentions this species as being present in El Salvador, but does not give specific records.

**8. *Acanthoscelidesguerrero* Johnson, 1983**

**Remarks.**Johnson (1990) mentions this species as being present in El Salvador, but does not give specific records.

**9. *Acanthoscelideshectori* Kingsolver, 1980**

**Remarks.** One specimen from El Salvador was intercepted at USDA Plant Quarantine, Washington DC (Kingsolver 1980).

**10. *Acanthoscelidesmacrophthalmus* (Schaeffer, 1907)**

**Published records.** LA LIBERTAD: San Andrés, 6/VI/1958 (Johnson 1979).

**11. *Acanthoscelidesmankinsi* Johnson, 1983**

**Remarks.**Johnson (1990) mentions this species as being present in El Salvador, but does not give specific records.

**12. *Acanthoscelidesmegacornis* Kingsolver, 1980**

**Published records.** SANTA ANA: P. N. Montecristo, 7–9/V/1958 (Kingsolver 1980).

**13. *Acanthoscelidesobvelatus* Bridwell, 1942**

**Remarks.**Berry (1959) mentions this species as being present in El Salvador, but does not give specific records.

**14. *Acanthoscelidespuelliopsis* Johnson, 1983**

**Remarks.**Johnson (1990) mentions this species as being present in El Salvador, but does not give specific records.

**15. *Acanthoscelidespuellus* (Sharp, 1885)**

**Remarks.**Mancía and Cortéz (1975) and Johnson (1990) mention this species as being present in El Salvador, but do not give specific records.

**16. *Acanthoscelidespusillimus* (Sharp, 1885)**

**Remarks.**Johnson (1990) mentions this species as being present in El Salvador, but does not give specific records.

**17. *Acanthoscelidesquadridentatus* (Schaeffer, 1907)**

**Remarks.**Johnson (1990) mentions this species as being present in El Salvador, but does not give specific records.

**18. *Acanthoscelidesrufovittatus* (Schaeffer, 1907)**

**Remarks.**Johnson (1990) mentions this species as being present in El Salvador, but does not give specific records.

**19. *Acanthoscelidestaboga* Johnson, 1983**

**Remarks.**Johnson (1990) mentions this species as being present in El Salvador, but does not give specific records.

**20. *Amblycerusmariae* Romero, Johnson & Kingsolver, 1996**

**Published records.** LA LIBERTAD: La Toma, 11/VI/1958 (Romero et al. 1996); SAN VICENTE: Santa Cruz Porrillo, 5/VI/1958.

**21. *Amblycerusmultiflocculus* Kingsolver, 1980**

**Published records.** SAN SALVADOR: Tonocatepeque, 20/VI/1958 (Kingsolver 1980).

**22. *Amblycerusscutellaris* (Sharp, 1885)**

**Remarks.**Romero et al. (1996) mentions this species as being present in El Salvador, but does not give specific records.

**23. *Amblycerusspondiae* Kingsolver, 1980**

**Published records.** LA LIBERTAD: Santa Tecla, 5/VI/1958; LA UNIÓN: Volcan Conchagua, 27–29/V/1958 (Kingsolver 1980); SAN SALVADOR: 9/VI/1958.

**24. *Amblycerusvegai* Kingsolver, 1996**

**Published records.** LA UNIÓN: Volcan Conchagua, 27–29/V/1958 (Kingsolver 1980).

**25. *Callosobruchusmaculatus* (Fabricius, 1775)**

**Remarks.**Mancía and Cortéz (1975) mention this species as being present in El Salvador, but do not give specific records. This species is native to the Oriental Region.

**26. *Callosobruchuschinensis* (Linnaeus, 1758)**

**Remarks.**Mancía and Cortéz (1975) mention this species as being present in El Salvador, but do not give specific records. This species is native to the Oriental Region.

**27. *Caryedesbrasiliensis* (Thunberg, 1816)**

**Published records.** SAN SALVADOR: Tonacatepeque, VI/1931 (Kingsolver and Whitehead 1974a).

**28. *Caryedesclitoriae* (Gyllenhal, 1839)**

**Published records.** Chalatenango: Quetzaltepeque, 19/VI/1963 (Kingsolver and Whitehead 1974a, published under name *C.confinis* (Sharp, 1885)).

**29. *Caryedeslongicollis* (Fahraeus, 1839)**

**Published records.** SANTA ANA: P. N. Montecristo, 23 km N of Metapan, 8–10/V/1971 (Kingsolver and Whitehead 1974a).

**30. *Caryedesquadridens* (Jekel, 1855)**

**Published records.** LA UNIÓN: Volcan Conchagua, 27–29/V/1958 (Kingsolver and Whitehead 1974a).

**31. *Caryobruchuscurvipes* (Latreille, 1811)**

**Published records.** LA LIBERTAD: Santa Tecla (Berry and Salazar 1957). SAN SALVADOR (Bridwell 1929).

**32. *Ctenocolumcrotonae* (Fahraeus, 1839)**

**Published records.** LA UNIÓN: Volcan Conchagua, 27–29/V/1958; SAN SALVADOR: 20/XII/1921–4/1/1922 (Kingsolver and Whitehead 1974b).

**33. *Gibbobruchuscristicollis* (Sharp, 1885)**

**Published records.** LA UNIÓN: Volcan Conchagua, 27–29/V/1958 (Kingsolver and Whitehead 1975b).

**34. *Gibbobruchusguanacaste* Kingsolver & Whitehead, 1975**

**Published records.** LA UNIÓN: Volcan Conchagua, 27–29/V/1958 (Kingsolver and Whitehead 1975b).

**35. Megacerus (Pachybruchus) bifloccosus (Motschulsky, 1874)**

**Published records.** LA UNIÓN: Volcan Conchagua, 27–29/V/1958 (Teran and Kingsolver 1977).

**36. *Megacerus* (*Serratibruchus***) ***cubiciformis* (Sharp, 1885)**

**Published records.** LA UNIÓN: Volcan Conchagua, 27–29/V/1958 (Teran and Kingsolver 1977).

**37. *Megasenniusmuricatus* (Sharp, 1885)**

**Published records.** LA LIBERTAD: Quetzaltepeque, 19/VI/1958; SAN SALVADOR: 5/VI/1958 (Whitehead and Kingsolver 1975a).

**38. *Meibomeusapicicornis* (Pic, 1933)**

**Published records.** LA LIBERTAD: San Andrés (Kingsolver et al. 1976). LA UNIÓN: Volcan Conchagua (Kingsolver et al. 1976). SAN SALVADOR: San Salvador, Tonocatepeque (Kingsolver et al. 1976).

**39. *Meibomeuscampbelli* Kingsolver & Whitehead, 1976**

**Published records.** LA LIBERTAD: Quezaltepeque (Kingsolver and Whitehead 1976).

**40. *Meibomeushowdeni* Kingslover & Whitehead, 1976**

**Published records.** SAN SALVADOR: San Salvador, Boquerón, 1800 m, nr. Santa Tecla, 2/V/1971, leg. F. Howden, (Kingsolver et al. 1976).

**41. *Meibomeussurrubresus* (Pic, 1933)**

**Published records.** El Salvador, without further data (Mancía and Cortéz 1975). LA LIBERTAD: La Libertad, Quezaltepeque, San Andrés; LA UNIÓN: Volcan Conchagua SAN SALVADOR: San Salvador; USULUTÁN: Santiago de Maria (Kingsolver et al. 1976).

**42. *Merobruchuscolumbinus* (Sharp, 1885)**

**Published records.** La Libertad, La Paz, San Salvador (Kingsolver 1988).

**43. *Merobruchuscristoensis* Kingsolver, 1988**

**Published records.** SANTA ANA: P. N. Montecristo, 23 km N of Metapan, 8–10/V/1971 (Kingsolver 1988).

**44. *Merobruchusknulli* (White, 1941)**

**Published records.** SAN VICENTE (Kingsolver 1988).

**45. *Mimosesteshumeralis* (Gyllenhal, 1833)**

**Published records.** SANTA ANA: 8/I/1922 (Kingsolver and Johnson 1978).

**46. *Mimosestesmimosae* (Fabricius, 1781)**

**Published records.** SAN SALVADOR: San Salvador, 24/V/1958 and 14/VI/1958 (Kingsolver and Johnson 1978).

**47. *Mimosestesnubigens* (Motschulsky, 1874)**

**Published records.** CUSCATLÁN: 6 mi S of Candeleria, 20/VI/1968; El Carmen, 27/V/1958; LA UNIÓN: 14 mi SW of La Union, 23/VI/1968 (Kingsolver and Johnson 1978).

**48. *Pygiopachymeruslineola* (Chevrolat, 1871)**

**Published records.** LA UNIÓN: La Unión, (Berry and Salazar 1957). SAN SALVADOR: 1920 (Kingsolver, 1970).

**Remarks.**Berry and Salazar (1957) reported this species under the name *Phelomerusaberrans* (Shary).

**49. *Senniusabbreviatus* (Say, 1824)**

**Published records.**Mancía and Cortéz (1975) mention this species (under the name *S.bivulneratus* (Horn, 1873)) as being present in El Salvador, but do not give specific records.

**50. *Senniusatripectus* Johnson & Kingsolver, 1973**

**Published records.** SANTA ANA: Metapán, P. N. Montecristo, 7–9/V/1958, leg. O.L. Cartwright, (Johnson and Kingsolver 1973).

**51. *Senniusdiscolor* (Horn, 1873)**

**Published records.**Mancía and Cortéz (1975) mention this species as being present in El Salvador, but do not give specific records.

**52. *Senniusfallax* (Boheman, 1839)**

**Published records.** LA LIBERTAD: Santa Tecla (Berry and Salazar 1957).

**Remarks.** In Berry and Salazar (1957) this species is given under the name *S.probus* which was synonymized with *S.fallax* in (Johnson and Kingsolver 1973). This species record of Berry was not included in the publication of Johnson and Kingslover (1973).

**53. *Senniuslebasi* (Fahraeus, 1839)**

**Remarks.**Johnson and Kingsolver (1973) mention this species (under the name *S.celatus* (Sharp, 1885)) as being present in El Salvador, but do not give specific records.

**54. *Senniusmorosus* (Sharp, 1885)**

**Published records.**Johnson and Kingsolver (1973) and Mancía and Cortéz (1975) mention this species as being present in El Salvador, but does not give specific records.

**55. *Senniusobesulus* (Sharp, 1885)**

**Published records.** LA UNIÓN: without further locality data (Johnson and Kingsolver 1973).

**56. *Senniusrufomaculatus* (Motschulsky, 1874)**

**Published records.**Johnson and Kingsolver (1973) mention this species as being present in El Salvador, but do not give specific records.

**57. *Statorlimbatus* (Horn, 1873)**

**Published records.** LA LIBERTAD: San Andrés, V/1958 (Johnson and Kingsolver 1976). LA UNIÓN: 14 mi SW La Unión, 23/VI/1968, Leg. C. D. Johnson (Johnson 1976).

**58. *Statorpruininus* (Horn, 1873)**

**Remarks.** Johnson (1976) and Johnson and Kingsolver (1976) mention this species as being present in El Salvador, but do not give specific records.

**59. *Statorsordidus* (Horn, 1873)**

**Remarks.** Johnson (1976) and Johnson and Kingsolver (1976) mention this species as being present in El Salvador, but does not give specific records.

**60. *Statorvachelliae* Bottimer, 1973**

**Remarks.**Johnson and Kingsolver (1976) mention this species as being present in El Salvador, but do not give specific records.

**61. *Zabroteschavesi* Kingsolver, 1980**

**Published records.** SAN SALVADOR: San Salvador, 14/VI/1958 (Kingsolver 1980).

**62. *Zabrotesinterstitialis* (Chevrolat, 1871)**

**Published records.** SAN SALVADOR: San Salvador, 22–26/VI/1958 (Kingsolver 1970b).

**63. *Zabrotessubfasciatus* (Boheman, 1833)**

**Published records.** SAN SALVADOR: San Salvador (Berry 1957).

**Remarks.**Kingsolver (1990) mentioned cosmopolitan distribution for this species but did not name individual contries, where the species was confirmed.

#### Subfamily Cassidinae

**1. *Anisostenafunesta* (Baly, 1885)**

**Published records.** SAN SALVADOR: San Salvador, 14/VI/1948, 9/VI/1958, 21/VI/1958, 25/VI/1958; SANTA ANA: Valiano (Staines 1994b).

**Specimens examined.** La Libertad: Santa Tecla, 28/IV/1960, 1 spec., J. Bechyné leg. (RBINS). MORAZÁN: Perguin, 22/VI/1959, 1 spec., J. Bechyné leg. (LSC).

**2. *Anisostenaperspicua* (Horn, 1883)**

**Published records.** LA LIBERTAD: Quezaltepeque, 500 m a.s.l., 19/VI/1963, 7–8/VII/1963, 15/VII/1963, 4/VIII/1963 (Staines 1994b).

**3. *Anisostenapilatei* (Baly, 1864)**

**Published records.** LA LIBERTAD: Quezaltepeque, 500 m a.s.l., 19/VI/1963, 5/VII/1963, 15/VII/1963; Santa Tecla, 4/VI/1959 (Staines 1994a).

**4. *Anisostenatrilineata* (Baly, 1864)**

**Published records.** SAN SALVADOR: San Salvador, 5/VI/1958, 21/VI/1958, 25/VI/1958 (Staines 1994a).

**Specimens examined.** SANTA ANA: Volcan San Diego, 22–24/VI/1959, 1 spec., J. Bechyné leg. (RBINS).

**5. *Baliosusfraternus* (Baly, 1885) new record**

**Specimens examined.** CuscatlÁn: Hacienda Colima, 12/VII/1959, 9 spec., 22/VII/1959, 47 spec., J. Bechyné leg. (RBINS, 10 LSC); La Libertad: San Andrés, 15/VI/1959, 1 spec., J. Bechyné leg. (RBINS); SANTA ANA: Volcan San Diego, 22–24/VI/1959, 1 spec., J. Bechyné leg. (RBINS).

**6. *Baliosusmarmoratus* (Baly, 1885)**

**Published records.** SAN VICENTE: Ichamichen, 150 m a.s.l., 5/IX/1958, 1 spec. (Uhmann 1961).

**Specimens examined.** La UniÓn: Cutuco, 2–3/VI/1959, 1 spec., J. Bechyné leg. (RBINS).

**7. *Brachycorynapumila* Guérin-Méneville, 1844**

**Published records.** SAN SALVADOR: Toma de Aguilares (Berry and Salazar 1957).

**Specimens examined.** San Salvador: San Salvador, 14–30/VI/1959, 1 spec., J. Bechyné leg. (RBINS).

**8. *Cephaloleiaruficollis* Baly, 1859**

**Published records.** CUSCATLÁN: El Rosario (Berry and Salazar 1957). LA LIBERTAD: Los Chorros, 4 km S of Santa Tecla, 13/V/1971 (Staines 1996).

**9. *Cephaloleiatenella* Baly, 1885**

**Published records.** SAN SALVADOR: San Salvador, 14/VI/1958, 21/VI/1958, 1 spec. (Staines 1996).

**10. *Chalepusacuticornis* (Chapuis, 1877)**

**Remarks.**Berry (1959) mentions this species as being present in El Salvador, but does not give specific records. Species of *Chalepus* are rather difficult for identification and thus presence of this species El Salvador must be verified.

**11. *Chalepusamabilis* Baly, 1885**

**Published records.** CUSCATLÁN: El Rosario (Berry and Salazar 1957). LA LIBERTAD: Los Chorros, 500 m a.s.l., 12/X/1958, 3 spec. (Uhmann 1961).

**12. *Chalepusbellulus* (Chapuis, 1877)**

**Published records.** SAN VICENTE: Santa Cruz Porrillo (Berry and Salazar 1957). SONSONATE: Icalo [sic.!; = Izalco], 400 m a.s.l., 28/IX/1958, 1 spec. (Uhmann 1961).

**Specimens examined.** SANTA ANA: Volcan San Diego, 22–24/VI/1959, 2 spec., 25/VI/1959, 1 spec., J. Bechyné leg. (RBINS, 1 LSC).

**13. *Chalepuspici* Descarpentries & Villiers, 1959**

**Specimens examined.** LA LIBERTAD: Santa Tecla (Berry and Salazar 1957); LA PAZ: Volcan San Vicente, Finca La Paz, 1/VIII/1959, 1 spec., 5–6/VIII/1959, 1 spec., J. Bechyné leg. (RBINS, LSC).

**Remarks.**Berry and Salazar (1957) published this species as *Chalepus* cf. *quadricostatus reductus* Pic. The name is a homonym and was replaced by *C.pici*. Occurrence of this species in El Salvador confirmed.

**Figure 2.**
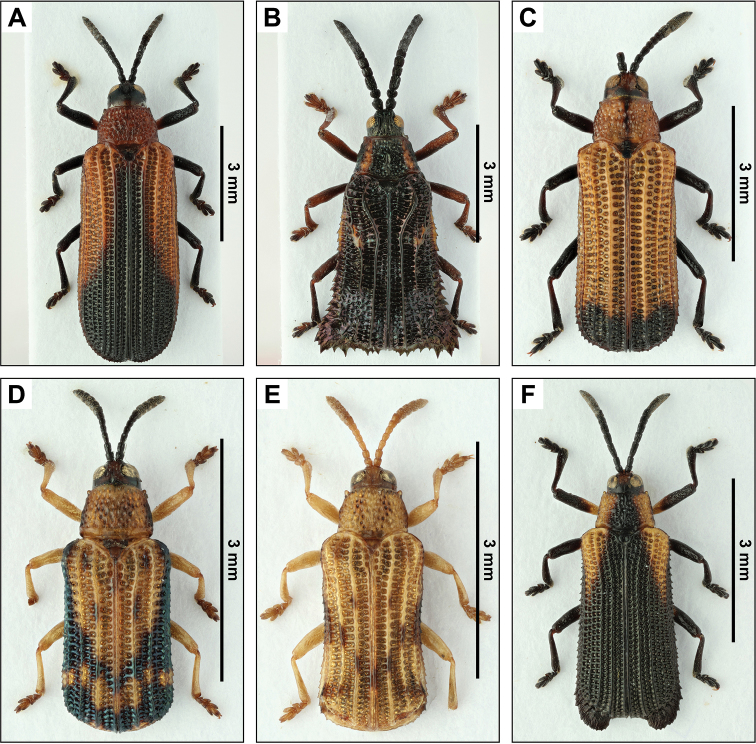
Dorsal pictures of some of the species of Cassidinae from El Salvador from the collections of the RBINS. **A***Chalepussimulatus***B***Euprionotagebieni***C***Octhispaatroterminata***D***Sumitrosisdistinctus***E***Sumitrosisfryi***F***Uroplatasculptilis*. High resolution images can be found at http://collections.naturalsciences.be/ssh-entomology.

**14. *Chalepussimilatus* Baly, 1885 new record (Fig. 2A)**

**Specimens examined.** AhuachapÁn: Apaneca, 14–15/VII/1959, 5 spec., J. Bechyné leg. (RBINS, 2 LSC); La UniÓn: Cutuco, 2–3/VI/1959, 12 spec., J. Bechyné leg. (RBINS, 4 LSC); SANTA ANA: Volcan San Diego, 22–24/VI/1959, 3 spec., J. Bechyné leg. (RBINS, 1 LSC).

**15. *Chalepusverticalis* (Chapuis, 1877) new record**

**Specimens examined.** SAN SALVADOR: El Salvador, 12/VII/1959, 1 spec., J. Bechyné leg. (LSC). La Libertad: Santa Tecla, 28/IV/1960, 1 spec., J. Bechyné leg. (RBINS).

**Remarks.**Berry (1959) published a record of *Chalepus* sp. probably *verticalis* without stating precise collecting data.

**16. Charidotella(s. str.)bifossulata (Boheman, 1855)**

**Remarks.**Berry (1959) mentions this species as being present in El Salvador, but does not give specific records. Occurrence of this species in El Salvador is probable.

**17. Charidotella(s. str.)egregia (Boheman, 1855)**

**Remarks.**Berry (1959) mentions this species as being present in El Salvador, but does not give specific records. Occurrence of this species in El Salvador is probable.

**18. Charidotella(s. str.)sexpunctata (Fabricius, 1781)**

**Published records.** LA LIBERTAD: Santa Tecla; Zaragoza, 1 spec.; SAN SALVADOR: Guazapa, 11/V/1960, 2 spec., J. Bechyné leg. (Borowiec 1996). SAN VICENTE: Santa Cruz Porrillo (Berry and Salazar 1957). SANTA ANA: Cerro Verde, 14/V/1976, 1 spec., A. Muyshondt leg.; Montecristo, Metapán, 23/V/1976, 1 spec., A. Muyshondt leg. (Borowiec 2009).

**Specimens examined.** UNKNOWN PROVINCE: Cafetalera, 14/VIII/1956, 4 spec. (SMTD).

**Remarks.**Berry and Salazar (1957) and Berry (1959) published this species under the name *Metrionatrisignata* (Boheman, 1855), which is considered a synonym of *C.sexpunctata*.

**19. Charidotella(s. str.)succinea (Boheman, 1855)**

**Published records.** El Salvador, without further data (Berry 1959 as Metrionacf.profligata Boheman, 1862); SANTA ANA: Montecristo, Metapán, 23/V/1976, 1 spec., A. Muyshondt leg. (Borowiec 2009: 638).

**20. Charidotella(s. str.)tuberculata (Fabricius, 1775)**

**Published records.** LA LIBERTAD: Los Chorros, Santa Tecla; SAN SALVADOR: Guazapa, 10/IX/1959, 3 spec., J. Bechyné leg. (Borowiec 1996); SONSONATE: Sonsonate (Berry and Salazar 1957).

**Remarks.** Borowiec (1996) recorded the species as new to El Salvador.

**21. ? Charidotella(s. str.)tumida (Champion, 1894)**

**Remarks.**Berry (1959) mentions this species as being present in El Salvador, but does not give specific records. The species is known so far only from Costa Rica and Panama, and its occurrence in El Salvador is not very probable.

**22. Charidotella(s. str.)virgulata (Boheman, 1855)**

**Specimens examined.** SANTA ANA: Cerro Verde, 1500 m, 7.ix.1958, 1 spec., Steinhausen leg. (DBET); SAN SALVADOR: San Salvador env., 1500 m, 6.ix.1958, 1 spec., Steinhausen leg. (DBET).

**Remarks.** Borowiec (1989) mentions this species as being present in El Salvador, but does not give specific records. The record was based on the first of the abovementioned specimens. The second specimen was originally identified as C. (Xenocassis) irazuensis (Champion, 1894), however, in fact belongs also to *C.virgulata* (L. Borowiec, pers. comm.). Therefore *C.irazuensis* does not occur in El Salvador.

**23. Charidotella (Chaerocassis) emarginata (Boheman, 1855)**

**Published records.** LA LIBERTAD: Santa Tecla (Berry and Salazar 1957).

**Remarks.** Occurrence of this species in El Salvador is probable.

**24. ? Charidotella (Xenocassis) cf.ambita (Champion, 1894).**

**Remarks.**Berry (1959) mentions this species as being present in El Salvador, but does not provide specific records. Certainly species of the subgenus Xenocassis Spaeth, 1936 must occur in El Salvador but so far formally none was recorded. The species are very similar to each to other and the identification is not easy without comparative material. Charidotella (X.) ambita is distributed in southern part of Central America (S Nicaragua to Panama), however, its occurrence in El Salvador cannot be excluded but is improbable.

**25. *Charidotisvitreata* (Perty, 1830)**

**Remarks.**Berry (1959) mentions this species as being present in El Salvador, but does not give specific records. Occurrence of this species in El Salvador is very probable.

**26. *Chelymorphacomata* Boheman, 1854**

**Published records.** El Salvador, without further data (Berry 1959 as Chelymorphacf.comata). SAN SALVADOR: Guazapa, 10/IX/1959, 1 spec., J. Bechyné leg. (Borowiec 1996: 158); SANTA ANA: Volcan San Diego, 22.–24/VI/1959, 1 spec., J. Bechyné leg. (Borowiec 2009).

**27. *Chelymorphagressoria* Boheman, 1862**

**Published records.** LA LIBERTAD: Comasagua, 1/VII/1959, 1 spec., J. Bechyné leg. (Borowiec 2009).

**28. *Chelymorphapubescens* Boheman, 1854**

**Published records.** El Salvador, without further data (Berry 1959, as *Clelymorpha* [sic!] cf.pubescens). CUSCATLÁN: El Rosario (Berry and Salazar 1957).

**Remarks.** Occurrence of this species in El Salvador is probable.

**29. Coptocycla(s. str.)sordida Boheman, 1855**

**Specimens examined.** UNKNOWN PROVINCE: Alomeya, 17/VIII/1920, 1 spec. (BMNH).

**30. Coptocycla (Psalidonota) leprosa Boheman, 1855**

**Published records.** El Salvador, without further data (Berry 1959). LA LIBERTAD: Los Chorros (Berry and Salazar 1957). SAN SALVADOR: Guazapa, 11/V/1960, 1 spec., J. Bechyné leg. (Borowiec 1996).

**31. *Deloyalafuliginosa* (Olivier, 1790)**

**Published records.** LA LIBERTAD: Cerro Litoral, 6/VI/1976, 1 spec., A. Muyshondt leg.; Zaragosa, 1 spec. (Borowiec 2009). SAN SALVADOR: Guazapa, 10/IX/1959, 6 spec., J. Bechyné leg. (Borowiec 1996; as *D.guttata* (Olivier, 1790)). SAN VICENTE: Santa Cruz Porrillo (Berry and Salazar 1957).

**Remarks.**Berry and Salazar (1957), Berry (1959) and Borowiec (1996) published this species under the name *D.guttata* (Olivier, 1790). Althought we have not examined the respective specimens, these records must belong to *D.fuliginosa* as *D.guttata* does not occur south of USA.

**32. ? *Deloyalalecontei* (Crotch, 1873)**

**Remarks.**Berry (1959) mentions this species as being present in El Salvador, but does not give specific records. Occurrence of this species in El Salvador is dubious as it is distributed in southern part of United States and north and central Mexico.

**33. *Deloyalazetterstedti* (Boheman, 1855)**

**Published records.** El Salvador, without further data (Berry 1959). LA LIBERTAD: C. Litoral, 6/VI/1976, 1 spec., A. Muyshondt leg. (Borowiec 2009). SAN SALVADOR: Guazapa, 11/V/1960, 1 spec., J. Bechyné leg. (Borowiec 1996).

**Remarks.**Berry (1959) mentioned both colour forms, with and without posterolateral spots on explanate margin of elytra. The other form was named *Coptocyclasallei* Boheman, 1862.

**34. *Euprionotaatterima* Guérin-Méneville, 1844**

**Published records.** LA LIBERTAD: Los Chorros, 500 m a.s.l., 12/X/1958, 1 spec. (Uhmann 1961).

**35. *Euprionotagebieni* (Uhmann, 1930) (Fig. 2B)**

**Published records.** SAN SALVADOR: San Salvador, 700 m a.s.l., 30/V/1956, 1 spec., E. Möhn leg. (Uhmann 1960).

**Specimens examined.** Chalatenango: La Paluma, 7–9/VII/1959, 1 spec., J. Bechyné leg. (RBINS); LA LIBERTAD: Hacienda Argentina, 4/IV/1960, 1 spec., J. Bechyné leg. (RBINS); Tamanique, 4/V/1960, 1 spec., J. Bechyné leg. (RBINS).

**36. *Heterispavinula* (Erichson, 1847) new record**

**Published records.** El Salvador, without further data (Berry 1959) under the name Uroplatacf.westwoodi Baly, 1885.

**Specimens examined.** LA LIBERTAD: Los Chorros, 29/VI/1959, 2 spec., J. Bechyné leg. (RBINS, LSC). LA PAZ: Volcan San Vicente, Finca La Paz, 1/VIII/1959, 2 spec., J. Bechyné leg. (RBINS). UsulutÁn: Jucuaran, 10–11/XI/1959, 1 spec., J. Bechyné leg. (RBINS),

**37. ? Helocassiscf.clavata (Fabricius, 1798)**

**Published records.** SAN SALVADOR: San Salvador (Berry and Salazar 1957).

**Remarks.** Record by Berry and Salazar (1957) probably belongs to *H.testudinaria* as typical *H.clavata* does not occur in tropical America. Moreover, *H.clavata* is not repeated in his second list (Berry 1959).

**38. *Helocassiscrucipennis* (Boheman, 1855)**

**Published records.** SONSONATE: San Julián (Berry and Salazar 1957).

**Remarks.** Occurrence of this species in El Salvador is very probable.

**39. *Helocassistestudinaria* (Boheman, 1855)**

**Published records.** El Salvador, without further data (Berry 1959). LA LIBERTAD: Los Chorros, 9/VI/1974, 1 spec., A. Muyshondt leg.; Zaragosa, 2 spec., A. Muyshondt leg. (Borowiec 2009).

**40. *Ischnocodiaannulus* (Fabricius, 1781)**

**Published records.** El Salvador, without further data (Berry 1959). AhuAchapÁn: Los Imposibles, 28/III/1976, 1 spec., A. Muyshondt leg. (Borowiec 2009).

**41. *Metrionellabilimeki* Spaeth, 1932**

**Published records.** SAN SALVADOR: Guazapa, 10/IX/1959, 1 spec., J. Bechyné leg. (Borowiec 1996).

**42. *Metrionellaerratica* (Boheman, 1855)**

**Published records.** SAN SALVADOR: Guazapa, 10/IX/1959, 1 spec., J. Bechyné leg. (Borowiec 1996).

**43. *Microctenochiraferranti* (Spaeth, 1926)**

**Published records.** El Salvador, without further data (Berry 1959). SAN SALVADOR: Guazapa, 11/V/1960, 5 spec., J. Bechyné leg. (Borowiec 1996).

**44. *Microctenochirahectica* (Boheman, 1855)**

**Published records.** El Salvador, without further data (Berry 1959). CUSCATLÁN: El Rosario (Berry and Salazar 1957). SAN SALVADOR: Guazapa, 11/V/1960, 1 spec., J. Bechyné leg. (Borowiec 1996).

**45. Microctenochiracf.plebeja (Boheman, 1855)**

**Remarks.**Berry (1959) mentions this species as being present in El Salvador, but does not give specific records. Its occurrence in El Salvador is very probable.

**46. *Microrhopalaperforata* Baly, 1864**

**Published records.** CUSCATLÁN: El Rosario (Berry and Salazar 1957). LA LIBERTAD: Los Chorros, 500 m a.s.l., 12/X/1958, 1 spec. (Uhmann 1961); Santa Tecla (Berry and Salazar 1957). SAN VICENTE: Ichamichen, 150 m a.s.l., 5/IX/1958, 1 spec.

**Specimens examined.** CuscatlÁn: Hacienda Colima, 12/VII/1959, 1 spec., J. Bechyné leg. (LSC); LA LIBERTAD: El Boquerón, 26/V/1960, 1 spec., J. Bechyné leg. (RBINS). SANTA ANA: Volcan San Diego, 15/VI/1959, 1 spec., 22–24/VI/1959, 1 spec., J. Bechyné leg. (RBINS).

**47. *Microrhopalapulchella* Baly, 1864**

**Published records.** El Salvador, without further data (Berry 1959). SAN SALVADOR: San Salvador env., 17/VIII/1958, 1 spec. (Uhmann 1961).

**48. *Octhispaatroterminata* Uhmann, 1943 new record (Fig. 2C)**

**Specimens examined.** LA LIBERTAD: El Boquerón, 10/VI/1959, 1 spec., J. Bechyné leg. (LSC); Tamanique, 4/V/1960, 1 spec., J. Bechyné leg. (RBINS). UsulutÁn: Jucuaran, 10.–11/XI/1959, 1 spec., J. Bechyné leg. (RBINS).

**Remarks.** The three specimens from El Salvador have the black stripe on their pronotum rather thin and only apical 1/5 of elytra black while the holotype of *O.atroterminata* has a thick medial stripe on pronotum and apical 1/3 of elytra black and the dark colouration extending along suture forwards. However, Salvadorian specimens show variability in black colouration: one has the pronotal stripe only slightly indicated; two have the apical black colouration on elytra rather emarginate near suture anteriorly while the third specimen has a clearly projecting black stripe along suture. Therefore, we consider these as intraspecific variability as other characters (e.g. general shape, punctation of elytra and pronotum) are similar to the holotype.

**49. *Octhispacentromaculata* (Chapuis 1877) new record**

**Specimens examined.** La Libertad: Santa Tecla, 28/IV/1960, 1 spec., J. Bechyné leg. (RBINS).

**50. *Octotomachampioni* Baly, 1885**

**Published records.** SAN VICENTE: Ichamichen, 150 m a.s.l., 17/V/1958, 17/VIII/1958, 5 spec. (Uhmann 1961).

**Specimens examined.** LA PAZ: Volcan San Vicente, Finca La Paz, 1/VIII/1959, 2 spec., J. Bechyné leg. (RBINS, LSC).

**51. *Octotomascabripennis* Guérin-Méneville, 1844**

**Published records.** San Salvador: San Salvador, 1953, x.1965 (Staines 1989).

**Specimens examined.** LA PAZ: Volcan San Vicente, Finca La Paz, 1/VIII/1959, 1 spec., J. Bechyné leg. (LSC). San Salvador: El Salvador, 12/VII/1959, 1 spec., J. Bechyné leg. (RBINS); San Salvador, 14.–30/VI/1959, 2 spec., J. Bechyné leg. (RBINS).

**52. *Oxychalepusalienus* (Baly, 1885)**

**Published records.** MORAZÁN: Perguin, 22/VI/1959; SANTA ANA: Metapán, Hacienda Montecristo, 2300 m a.s.l., 12/III/1972 (Staines 2010).

**53. *Oxychalepusanchora* (Chapuis, 1877)**

**Published records.** La Libertad: La Libertad, 10 m a.s.l., 15/XII/1972; San Salvador: Santa Tecla, 9/IV/1957 (Staines 2010).

**54. *Oxychalepusbalyanus* (Weise, 1911)**

**Published records.** SAN VICENTE: Ichamichen, 150 m a.s.l., 5/IX/1958, 2 spec. (Uhmann 1961: 20); Sonsonate: Ishuatan, 15/IX/1958 (Staines 2010).

**Specimens examined.** La Libertad: Comasagna, 3/VII/1959, 1 spec., J. Bechyné leg. (RBINS).

**55. *Parorectisrugosa* (Boheman, 1854)**

**Published records.** LA LIBERTAD: Los Chorros, 9/VI/1974, 1 spec., A. Muyshondt leg. (Borowiec 2009); SAN SALVADOR: Guazapa, 11/V/1960, 1 spec., J. Bechyné leg. (Borowiec 1996).

**56. *Pentispachevrolati* (Chapuis, 1877) new record**

**Specimens examined.** AhuachapÁn: Apaneca, 14–15/VII/1959, 2 spec., J. Bechyné leg. (RBINS, 1 LSC); SANTA ANA: Cerro Verde, 16/V/1960, 7 spec., J. Bechyné leg. (RBINS, 1 LSC).

**57. *Pentispafairmairei* (Chapuis, 1877)**

**Published records.** CUSCATLÁN: El Rosario (Berry and Salazar 1957). SAN SALVADOR: San Salvador env., 17/VIII/1958, 5 spec. (Uhmann 1961).

**Specimens examined.** Chalatenango: La Palma, 7–9/VII/1959, 1 spec., J. Bechyné leg. (RBINS). La Libertad: Comasagna, 3/VII/1959, 2 spec., J. Bechyné leg. (RBINS); El Boquerón, 10/VI/1959, 1 spec., 25/V/1960, 1 spec., J. Bechyné leg. (LSC); Santa Tecla, 28/IX/1959, 1 spec., J. Bechyné leg. (RBINS); Tamanique, 4/V/1960, 4 spec., J. Bechyné leg. (RBINS, 2 LSC). LA PAZ: Volcan San Vicente, Finca La Paz, 1/VIII/1959, 2 spec., J. Bechyné leg. (RBINS). UsulUtÁn: Alegria, 22/II/1960, 1 spec., J. Bechyné leg. (RBINS).

**Remarks.**Berry and Salazar (1957) published this species as *Uroplatapairmairei* Chap., which is a wrong spelling.

**58. *Pentispamelanura* (Chapuis, 1877)**

**Published records.** LA LIBERTAD: Boqueron, 1700 m a.s.l., 11/X/1958, 2 spec. (Uhmann 1961).

**59. *Physonotaalutacea* Boheman, 1854**

**Published records.** SONSONATE: San Julián, Sonsonate (Berry and Salazar 1957).

**Specimens examined.** LA LIBERTAD: La Toma, 16/V/1958, 1 spec., O. L. Cartwright leg. (USNM). LA PAZ: Olocuilta, 200 m a.s.l., 19/IX/1958, 1 spec., Steinh. leg. (DBET). LA UNION: La Union, 2/X/1924, 1 spec., K. A. Salman leg. (USNM), 10/V/1954, 10 spec., P. A. Berry leg. (USNM), 15/VI/1954, 1 spec., C. A. leg. (USNM). San Salvador: Lihuatan, 25/V/1960, 1 spec. (DBET); San Salvador, 1929, 2 spec., S. Calderon leg. (USNM). SAN VICENTE: Cuscutlán Bridge on Lempa River, 10/V/1954, 2 spec., P. A. Berry leg. (USNM). Sonsonate: Izalco, 1/IX/1905, 1 spec., F. Knab leg. (USNM); Sonsonate, 24/VIII/1905, 1 spec., F. Knab leg. (USNM). USULUTÁN: Volcan Conchagua, 27–29/V/1958, 1 spec., O. L. Cartwright leg. (USNM).

**60. *Physonotaattenuata* Boheman, 1854**

**Published records.** SONSONATE: Sonsonate (Berry and Salazar 1957).

**Remarks.** Occurrence of this species in El Salvador should be verified as species of *Physonota* Boheman, 1854 are rather difficult for identification.

**61. *Physonotacitrina* Boheman, 1854**

**Published records.** SANTA ANA: Montecristo, Metapán, 23/V/1976, 3 spec., A. Muyshondt leg. (Borowiec 2009).

**62. ? Physonotacf.eucalypta Boheman, 1862**

**Remarks.**Berry (1959) mentions this species as being present in El Salvador, but does not give specific records. Occurrence of this species in El Salvador is possible. On the other hand identification of species of *Physonota* Boheman, 1854 is rather difficult and the record could belong to another species, e.g. *P.limoniata* Boheman, 1862.

**63. *Physonotagigantea* Boheman, 1854**

**Published records.** SONSONATE: Acajutla (Champion 1894), Sonsonate (Berry and Salazar 1957).

**Specimens examined.** SANTA ANA: Volcan San Diego, 22–24/VI/1959, 1 spec., J. Bechyné leg. (DBET).

**64. *Physonotalimoniata* Boheman, 1862**

**Published records.** LA LIBERTAD: Jayaque, 1/VI/1975, 1 spec., A. Muyshondt leg. (Borowiec 2009).

**65. *Platocthispafulvescens* (Baly, 1886)**

**Published records.** LA LIBERTAD: Sitio del Niño, 9/V/1956, 1 spec., E. Möhn leg. (Uhmann 1960).

**66. ? *Stolasnigrolineata* (Champion, 1893)**

**Remarks.**Berry (1959) mentions this species as being present in El Salvador, but does not give specific records. The species is given under genus, *Championaspis* Spaeth, 1913. The species is so far known only from high mountains in Costa Rica and Panama, therefore its occurrence in El Salvador is very questionable. On the other hand it is very characteristic species, but there is *Hilarocassisexlamations* (Linnaeus, 1767) occuring in Central America, which has also thin black lines on elytra.

**67. *Sumitrosisdistinctus* (Baly, 1885) new record (Fig. 2D)**

**Specimens examined.** La UniÓn: Cutuco, 2–3/VI/1959, 1 spec., J. Bechyné leg. (LSC). SANTA ANA: Volcan San Diego, 25/VI/1959, 1 spec., J. Bechyné leg. (RBINS).

**Remarks.**Berry (1959) mentioned *Anoptilis* sp. probabl. *distincta* Baly as being present in El Salvador, but does not give specific records. Occurrence of *S.distinctus* in El Salvador confirmed.

**68. *Sumitrosisfryi* (Baly, 1885) new record (Fig. 2E)**

**Specimens examined.** AhuachapÁn: Los Ausoles, 26/I/1960, 1 spec., J. Bechyné leg. (RBINS). LA LIBERTAD: El Boquerón, 10/VI/1959, 1 spec., J. Bechyné leg. (LSC).

**69. *Uroplatafusca* Chapuis, 1877**

**Published records.** SONSONATE: Izalco, 400 m a.s.l., 28/IX/1958, 1 spec. (Uhmann 1961).

**70. *Tapinaspiswesmaeli* (Boheman, 1855)**

**Published records.** El Salvador, without further data (Berry 1959). SANTA ANA: Lago de Coatepeque, 19/VII/????, C. F. & S. Hevel leg. (Chaboo 2002).

**71. *Uroplatasculptilis* Chapuis, 1877 new record (Fig. 2F)**

**Specimens examined.** LA PAZ: Volcan San Vicente, Finca La Paz, 1/VIII/1959, 1 spec., J. Bechyné leg. (RBINS). San Salvador: San Salvador, 14–30/VI/1959, 1 spec., J. Bechyné leg. (RBINS).

**72. Xenochalepus (Neochalepus) contubernalis (Baly, 1885)**

**Published records.** SAN SALVADOR: San Salvador env., 17/VIII/1958, 3 spec. (Uhmann 1961).

**Specimens examined.** LA LIBERTAD: Tamanique, 4/V/1960, 6 spec., J. Bechyné leg. (RBINS, 2 LSC). SAN SALVADOR: San Salvador, 19/VI/1959, 1 spec., J. Bechyné leg. (LSC). SANTA ANA: Cerro Verde, 15/II/1960, 1 spec., J. Bechyné leg. (RBINS); Volcan San Diego, 22–24/VI/1959, 4 spec., J. Bechyné leg. (RBINS, 1 LSC).

**73. Xenochalepus(s. str.)omogerus (Crotch, 1873)**

**Published records.** El Salvador, without further data (Berry 1959). LA LIBERTAD: Santa Tecla (Berry and Salazar 1957). SAN SALVADOR: San Salvador env., 17/VIII/1958, 3 spec. (Uhmann 1961).

**Specimens examined.** AhuachapÁn: Apaneca, 14.–15/VII/1959, 5 spec., 15/VII/1959, 1 spec., J. Bechyné leg. (RBINS, 2 LSC). CuscatlÁn: Hacienda Colima, 22/VII/1959, 1 spec., J. Bechyné leg. (RBINS). LA PAZ: Volcan San Vicente, Finca La Paz, 1/VIII/1959, 1 spec., J. Bechyné leg. (LSC). SANTA ANA: Volcan San Diego, 22–24/VI/1959, 1 spec., J. Bechyné leg. (LSC).

**74. Xenochalepus(s. str.)rufithorax (Baly, 1885)**

**Published records.** CUSCATLÁN: El Rosario (Berry and Salazar 1957). SAN SALVADOR: San Salvador env., 20/IX/1958, 1 spec. (Uhmann 1961: 20).

**Specimens examined.** SAN SALVADOR: El Salvador, 12/VII/1959, 2 spec., J. Bechyné leg. (RBINS, LSC).

**Remarks.**Berry and Salazar (1957) published this species under the name *Xenochalepusrufithoracesanguineus* Baly, which is wrong spelling. He must have meant *X.sanguinosus* (Baly, 1885), which is considered a synonym of *X.rufithorax*.

#### Subfamily Chrysomelinae

**1. *Calligraphaargus* Stål, 1859**

**Published records.** AHUACHAPÁN: Apaneca, 4500 ft, 7–12/IX/2002, D. Marqua, Calligraphaargus Stål J. Gómez-Zurita det. 2011 (Gómez-Zurita 2015). LA LIBERTAD: Tamanique, 1000 m, 5/XII/1971, S. & L. Steinhausen, Calligraphaargus Stål J. Gómez-Zurita det. 2011 (Gómez-Zurita 2015). LA UNIÓN: No. 444–7813, 15/VI/54, M.S.V. (Gómez-Zurita 2015). Volcan Conchagua, 27–29/V/1958, O.L. Cartwright, Calligraphaargus Stål J. Gómez-Zurita det. 2011 (Gómez-Zurita 2015). SANTA ANA: Metapán, No. 444–2806, 5/VII/1954, M.S.V. (Gómez-Zurita 2015).

**Remarks.**Bechyné and Bechyné (1965a) also report *C.argus* from El Salvador, but do not state specific localities.

**Figure 3.**
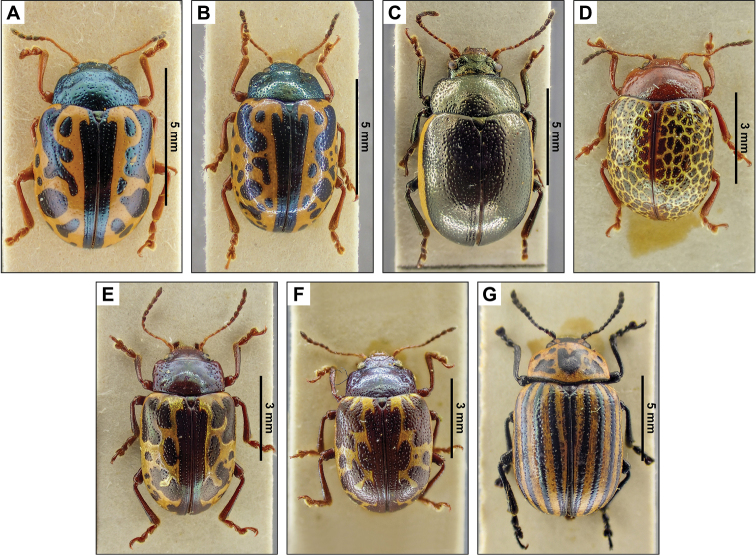
Dorsal pictures of the species of Chrysomelinae from El Salvador currently present in the collections of the RBINS. **A***Calligraphabajula***B***C.fulvipes***C**C. (Zygospila) championi**D**C. (Zygospila) guttulosa**E**C. (Zygospila) piceicollis**F**C. (Zygospila) signatipennis**G***Leptinotarsaundecimlineata*. High resolution images can be found at http://collections.naturalsciences.be/ssh-entomology.

**2. *Calligraphabajula* Stål, 1860 new record (Fig. 3A)**

**Specimens examined.** SAN SALVADOR: El Boquerón, 3/VII/1959, Leg. J. Bechyné, Det. J. Gómez-Zurita, 1 spec. (RBINS).

**Remarks.** For a full inventory of the *Calligrapha* spec. in the RBINS collections, one should consult Merckx et al. (2018), where the records cited here from El Salvador are also included.

**3. *Calligraphadiversa* Stål, 1859**

**Published records.** SANTA ANA: No. 444.2813, 5/VII/1954, Col. M.S.V., Calligraphadiversa Stål J. Gómez-Zurita det. 2011 (Gómez-Zurita 2015).

**4. *Calligraphafulvipes* Stål, 1859 (Fig. 3B)**

**Published records.** CUSCATLÁN: El Rosario (Berry 1957). SAN SALVADOR: 10/VI/1951 (Bechyné 1954); Volcan Santa Ana, 1600–1700 m, 3/VIII/1951 (Bechyné 1954).

**Specimens examined.** SAN SALVADOR: El Boquerón, 3/VII/1959, Leg. J. Bechyné, Det. J. Bechyné, 40 spec. (RBINS).

**Remarks.**Bechyné and Bechyné (1965a) also report *C.fulvipes* from El Salvador, but do not state specific localities. Most possibly this originates from the localities from Bechyné (1954) which are cited here. For a full inventory of the *Calligrapha* spec. in the RBINS collections, one should consult Merckx et al. (2018), where the records cited here from El Salvador are also included.

**5. *Calligraphamultiguttata* Stål, 1859**

**Published records.** AHUACHAPÁN: Apaneca, MIZA0040488, 13.860000°N, 89.801110°W, 17/VIII/1959, J. Bechyné & B. Bechyné coll., Calligraphamultiguttata St. J. Bechyné det. (Gómez-Zurita 2016).

**6. *Calligraphanupta* Stål, 1859 new record**

**Specimens examined.** SAN SALVADOR: El Boquerón, 3/VII/1959, Leg. J. Bechyné, Det. J. Gómez-Zurita, 1 spec. (RBINS).

**Remarks.** For a full inventory of the *Calligrapha* species in the RBINS collections, one should consult Merckx et al. (2018), where the records cited here from El Salvador are also included.

**7. *Calligrapharamulifera* Stål, 1859**

**Published records.** CUSCATLÁN: Rosario, Cuzcatlan, n°631–39, 17/VII/1955, Col. M.S.V. (Gómez-Zurita 2015). LA LIBERTAD: Los Chorros (Berry 1957); Los Chorros, 700 m, 4/VI/1972, S. & L. Steinhausen, *Calligrapharamulifera* Stål J. Gómez-Zurita det. 2011 (Gómez-Zurita 2015). SAN SALVADOR: 25/II/1920, E.S.C.A., K.A. Salman collector, *Calligrapharamulifera* Stål EAC’27 (Gómez-Zurita 2015). SANTA ANA: San Isidro, Cerro Verde 1000 m, 2/VI/1972, S. & L. Steinhausen, 2563, *Calligrapharamulifera* Stål J. Gómez-Zurita det. 2011 (Gómez-Zurita 2015). UNKNOWN PROVINCE: Landaverde, S. & L. Steinhausen, *Calligrapharamulifera* Stål Det. J. Watts 1993 (Gómez-Zurita 2015). Servicios Técnicos Cafetalería, El Salvador C.A. [one with: 14–E–42], (Gómez-Zurita 2015).

**8. *Calligraphasuboculata* Stål, 1859**

**Published records.** SANTA ANA: San Isidro, Cerro Verde, 1000 m, 11/VI/1972, S. & L. Steinhausen coll., *Calligraphasuboculata* Stål J. Gómez-Zurita det. 2011 (Gómez-Zurita, 2018).

**9. Calligrapha (Zygospila) bigenera (Stål, 1859)**

**Published records.** LA LIBERTAD: Santa Tecla, (Berry 1957).

**10. Calligrapha (Zygospila) championi (Jacoby, 1879) new record (Fig. 3C)**

**Specimens examined.** SAN SALVADOR: 19/VI/1959, Leg. J. Bechyné, Det. J. Bechyné, 4 spec., (RBINS)

**11. Calligrapha (Zygospila) dulcis (Stål, 1859)**

**Remarks.**Berry (1959) mentions this species as being present in El Salvador, but does not give specific records.

**12. Calligrapha (Zygospila) guttulosa (Stål, 1859) new record (Fig. 3D)**

**Specimens examined.** SANTA ANA: Cerro Verde, 16/V/1960, Leg. J. Bechyné, Det. J. Bechyné, 22 spec. (RBINS).

**13. Calligrapha (Zygospila) piceicollis (Stål, 1859) (Fig. 3E)**

**Published records.** LA LIBERTAD: Santa Tecla (Berry 1957). SAN SALVADOR: La Toma de Aguilares (Berry 1957).

**Specimens examined.** LA LIBERTAD: Hacienda Chanmico, 20/VI/1960, Leg. J. Bechyné, Det. J. Bechyné, 8 spec. (RBINS).

**14. Calligrapha (Zygospila) signatipennis (Stål, 1859) (Fig. 3F)**

**Published records.** SAN SALVADOR: 23/IV/1951 and 11/VII/1951, (Bechyné 1954).

**Specimens examined.** SAN SALVADOR: El Boquerón, 9/V/1960, Leg. J. Bechyné, Det. J. Bechyné, 5 spec. (RBINS).

**15. *Chrysomeladepressa* Suffrian, 1858**

**Published records.** SAN SALVADOR: San Salvador (Berry 1957).

**16. *Leptinotarsaflavitarsisflavitarsis* Guérin, 1855**

**Published records.** CUSCATLÁN: Volcan San Vicente, Finca El Carmen, 1300 m, 11–16/VI/1951 (Bechyné 1954). Hacienda Buena Vista, 1200 m, Volcan Izalco, 26/VII/1951 (Bechyné 1954). LA LIBERTAD: Los Chorros (Berry 1957).

**17. *Leptinotarsaundecimlineata* (Stål, 1859) new record (Fig. 3G)**

**Specimens examined.** SANTA ANA: Cerro Verde, 3/I/1960, Leg. J. Bechyné, Det. J. Bechyné, 28 spec. (RBINS).

**18. *Plagioderaaeneiventris* Stål, 1860**

**Published records.** CUSCATLÁN: El Rosario, (Berry, 1957).

**19. *Stilodesatromaculata* (Stål, 1859)**

**Published records.** LA LIBERTAD: Los Chorros (Berry 1957).

**Remarks.**Berry and Salazar (1957) mentions this species under its synonym *Deuterocamptaatromaculata*.

#### Subfamily Cryptocephalinae

**1. Babia(s. str.)quadriguttata Lacordaire, 1848**

**Published records.** LA LIBERTAD: Santa Tecla (Berry and Salazar 1957).

**2. *Chlamisusmaculipes* (Chevrolat, 1835)**

**Published records.** CUSCATLÁN: El Rosario (Berry and Salazar 1957).

**3. *Chlamisuspardalis* (Lacordaire, 1848)**

**Remarks.**Berry (1959) mentions this species as being present in El Salvador, but does not give specific records.

**4. *Cryptocephalustrizonatus* Suffrian, 1858**

**Published records.** LA LIBERTAD: Santa Tecla (Berry and Salazar 1957).

**5. *Griburiusalbilabris* (Suffrian, 1852)**

**Published records.** CUSCATLÁN: El Rosario (Berry and Salazar 1957).

**6. *Lexiphanesbimaculatus* (Jacoby, 1880)**

**Published records.** CUSCATLÁN: El Rosario (Berry and Salazar 1957).

**7. *Megalostomisdimidiata* Lacordaire, 1848**

**Remarks.**Berry (1959) mentions this species as being present in El Salvador, but does not give specific records.

**8. *Megalostomispyropyga* Lacordaire, 1848**

**Remarks.**Berry (1959) mentions this species as being present in El Salvador, but does not give specific records.

#### Subfamily Eumolpinae

**Figure 4.**
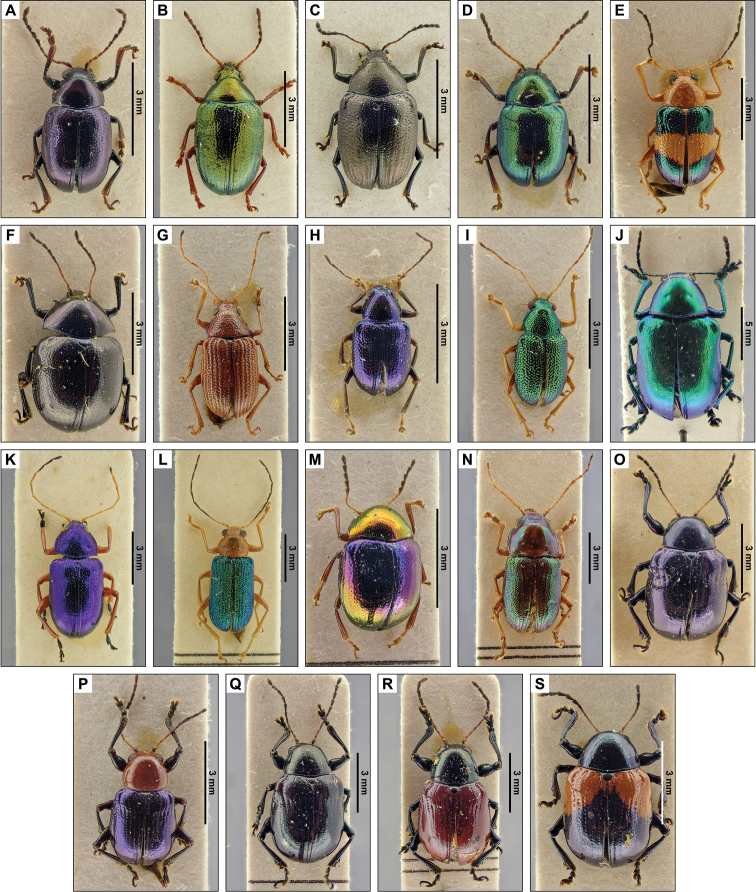
Dorsal pictures of the species of Eumolpinae from El Salvador currently present in the collections of the RBINS. Pictures of *nomina nuda* spec.s are not depicted. **A***Brachypnoeacretifera***B***B.lateralislateralis***C***B.lefeivreilefeivrei***D***B.viridis***E***Chalcophanacincta***F***Chrysodinopsiscupriceps***G***Colaspisfreyi***H***C.inconstans***I***C.lebasi***J***Eumolpusrobustus***K***Nodocolaspisimpressa***L***Prionoderahirtipennis***M***Spintherophytacorusca***N***Talurusrugosus***O***Typophoruslimbatus***P***Typophorusmexicanus***Q***Typophorusnigritusobliquus* var. a **R***Typophorusnigritusobliquus* var. b **S***Typophorusnigritusobliquus* var. c. High resolution images can be found at http://collections.naturalsciences.be/ssh-entomology.

**1. *Brachypnoeacretifera* (Lefèvre, 1875) new record (Fig. 4A)**

**Specimens examined.** SANTA ANA: Cerro Verde, 16/V/1960, Leg. J. Bechyné, Det. J. Bechyné, 61 spec. (RBINS).

**2. *Brachypnoealateralislateralis* (Jacoby, 1881) new record (Fig. 4B)**

**Specimens examined.** SANTA ANA: Cerro Verde, 16/V/1960, Leg. J. Bechyné, Det. J. Bechyné, 39 spec. (RBINS).

**3. *Brachypnoealefevreilefevrei* (Jacoby, 1878) new record (Fig. 4C)**

**Specimens examined.** SANTA ANA: Cerro Verde, 16/V/1960, Leg. J. Bechyné, Det. J. Bechyné, 21 spec. (RBINS).

**4. *Brachypnoeaviridis* (Jacoby, 1878) new record (Fig. 4D)**

**Specimens examined.** SAN SALVADOR, 23/V/1960, Leg. J. Bechyné, Det. J. Bechyné, 48 spec. (RBINS).

**5. *Chalcophanacincta* Harold, 1874 (Fig. 4E)**

**Published records.** CUSCATLÁN: El Rosario (Berry 1957).

**Specimens examined.** LA LIBERTAD: Comasagua, 3/VII/1959, Leg. J. Bechyné, Det. J. Bechyné, “No Type!”, 4 spec. (RBINS). SAN SALVADOR: 23/IV/1960, Leg. J. Bechyné, Det. J. Bechyné, 41 spec. (RBINS), 8/IV/1960, Leg. J. Bechyné, Det. J. Bechyné, 12 spec. (RBINS).

**6. *Chrysodinopsiscupriceps* Lefèvre, 1877 new record (Fig. 4F)**

**Specimens examined.** SAN SALVADOR: 24/V/1960, Leg. J. Bechyné, Det. J. Bechyné, 14 spec. (RBINS).

**7. *Colaspisfreyi* Bechyné, 1950 new record (Fig. 4G)**

**Specimens examined.** SAN SALVADOR: 10/IX/1959, Leg. J. Bechyné, Det. J. Bechyné, 11 spec. (RBINS), 11/V/1960, Leg. J. Bechyné, Det. J. Bechyné, 12 spec. (RBINS), 19/VI/1959, Leg. J. Bechyné, Det. J. Bechyné, 10 spec. (RBINS), 9/V/1960, Leg. J. Bechyné, Det. J. Bechyné, 17 spec. (RBINS).

**Remarks.** In the RBINS, these spec. are labeled as *Maecolaspisfreyi*. However, all species within *Maecolaspis* Bechyné, 1950 have been placed into *Colaspis* Fabricius, 1801; see Flowers (1996).

**8. *Colaspisimpressa* Lefèvre, 1877**

**Published records.** CUSCATLÁN: El Rosario (Berry 1957).

**9. *Colaspisinconspicua* Jacoby 1890**

**Published records.** CUSCATLÁN: El Rosario (Berry 1957).

**10. *Colaspisinconstans* (Lefèvre, 1878) new record (Fig. 4H)**

**Specimens examined.** SAN SALVADOR: Guazapa, 11/V/1960, Leg. J. Bechyné, Det. J. Bechyné, 14 spec. (RBINS).

**Remarks.** In the RBINS, these spec. are labeled as *Maecolaspisinconstans*. However, all species within *Maecolaspis* Bechyné, 1950 have been placed into *Colaspis* Fabricius, 1801; see Flowers (1996).

**11. *Colaspislebasi* (Lefèvre, 1878) (Fig. 4I)**

**Published records.** SAN SALVADOR: 10/VI/1951 (Bechyné 1954).

**Specimens examined.** SAN SALVADOR: 19/VI/1959, Leg. J. Bechyné, Det. J. Bechyné, 9 spec. (RBINS).

**Remarks.** In the RBINS, these spec. are labeled as *Maecolaspislebasi*. However, all species within *Maecolaspis* Bechyné, 1950 have been placed into *Colaspis* Fabricius, 1801; see Flowers (1996). Bechyné (1955) also states El Salvador under distribution of *C.lebasi* but does not add additional records.

**12. *Colaspismelancholica* Jacoby, 1881**

**Remarks.**Flowers (1996) states El Salvador under this species distribution, but does not give specific localities.

**13. *Colaspissuturalis* Lefèvre, 1878**

**Published records.** SAN SALVADOR: Toma de Aguilares (Berry 1957).

**14. *Colaspiszilchi* (Bechyné, 1954)**

**Published records.** SAN SALVADOR: 30/IV–5/V/1951 (Bechyné 1954).

**Specimens examined.** SAN SALVADOR: 19/VI/1959, Leg. J. Bechyné, Det. J. Bechyné, 21 spec. (RBINS).

**Remarks.** These spec. are labeled as *Maecolaspiszilchi*. However, all species within *Maecolaspis* Bechyné, 1950 have been placed into *Colaspis* Fabricius, 1801; see Flowers (1996).

**15. *Deuteronodasuturalissuturalis* Lefèvre, 1878**

**Remarks.**Bechyné and Bechyné (1965b) state El Salvador under this species distribution, but do not give any specific localities.

**16. *Eumolpusrobustus* (Horn, 1885) new record (Fig. 4J)**

**Specimens examined.** SAN SALVADOR: 23/V/1960, Leg. J. Bechyné, Det. J. Bechyné, 4 spec. (RBINS).

**17. *Fidiaunistriata* Jacoby, 1882**

**Remarks.**Berry (1959) mentions this species as being present in El Salvador, but does not give specific records.

**18. *Freudeitamelancholica* (Jacoby, 1881)**

**Remarks.**Bechyné and Bechyné (1969b) state El Salvador under this species distribution, but do not give any specific localities.

**19. *Glyptoscelischontalensis* Jacoby, 1882**

**Remarks.**Flowers (1996) states El Salvador under this species distribution, but does not give specific localities.

**20. *Habrophoramaculipennis* Jacoby, 1882**

**Remarks.**Berry (1959) mentions this species as being present in El Salvador, but does not give specific records.

**21. *Metachromavittipennis* Blake, 1970**

**Remarks.**Flowers (1996) states El Salvador under this species distribution, but does not give specific localities.

**22. *Nodocolaspisimpressa* (Lefèvre, 1877) new record (Fig. 4K)**

**Specimens examined.** LA LIBERTAD: Hacienda Argentina, 11/IX/1959, Leg. J. Bechyné, Det. J. Bechyné, 5 spec. (RBINS).

**Remarks.** In Flowers (1996), this species is listed under *Colaspis* Fabricius, 1801. However, *Nodocolaspis* Bechyné, 1949 is not listed here as a synonym of *Colaspis*. The correct placement of this species thus remains uncertain for the authors.

**23. *Prionoderahirtipennis* Jacoby, 1881 new record (Fig. 4L)**

**Specimens examined.** SAN SALVADOR: El Boquerón, 25/V/1960, Leg. J. Bechyné, Det. J. Bechyné, 24 spec. (RBINS).

**24. *Promecosomanobilitatum* Lefèvre, 1877**

**Published records.** SAN SALVADOR: 30/IV–5/V/1951 (Bechyné 1954).

**25. *Spintherophytacorrusca* (Lefèvre, 1877) new record (Fig. 4M)**

**Specimens examined.** SAN SALVADOR: El Boquerón, 25/IV/1960, Leg. J. Bechyné, Det. J. Bechyné, 15 spec. (RBINS), 24/V/1960, Leg. J. Bechyné, Det. J. Bechyné, 15 spec. (RBINS).

**26. *Talurusrugosus* (Jacoby, 1882) (Fig. 4N)**

**Published records.** CUSCATLÁN: El Rosario; SONSONATE: Santa Cruz Porrillo (Berry 1957).

**Specimens examined.** LA UNIÓN: Cutuco, 3/VI/1959, Leg. J. Bechyné, Det. J. Bechyné, 32 spec. (RBINS). SAN SALVADOR: 19/VI/1959, Leg. J. Bechyné, Det. J. Bechyné, 26 spec. (RBINS), 23/V/1960, Leg. J. Bechyné, Det. J. Bechyné, 1 spec. (RBINS).

**27. *Typophoruslimbatus* Jacoby, 1891 new record (Fig. 4O)**

**Specimens examined.** LA LIBERTAD: Hacienda Argentina, 19/V/1960, Leg. J. Bechyné, Det. J. Bechyné, 44 spec. (RBINS).

**28. *Typophorusmexicanus* (Jacoby, 1882) new record (Fig. 4P)**

**Specimens examined.** SAN SALVADOR: El Boquerón, 9/V/1960, Leg. J. Bechyné, Det. J. Bechyné, 48 spec. (RBINS).

**29. *Typophorusnigritusobliquus* Baly, 1859 new record (Fig. 4Q,R,S)**

**Specimens examined.** SAN SALVADOR: Cartagena, 19/V/1959, Leg. J. Bechyné, Det. J. Bechyné, 24 spec. (RBINS), 23/V/1960, Leg. J. Bechyné, Det. J. Bechyné, 26 spec. (RBINS).

#### Subfamily Galerucinae – Alticini

**1. *Acallepitrixanila* Bechyné & Bechyné, 1963**

**Published records.** LA LIBERTAD: Los Chorros, 25/VI/1959 (Bechyné and Bechyné 1963).

**2. *Acallepitrixclypeataheteronitens* Bechyné & Bechyné, 1963**

**Published records.** MORAZÁN: Perquín, 22/IX/1959 (Bechyné and Bechyné 1963). SAN SALVADOR: Capital, 27/IX/1959 (Bechyné and Bechyné 1963).

**Figure 5.**
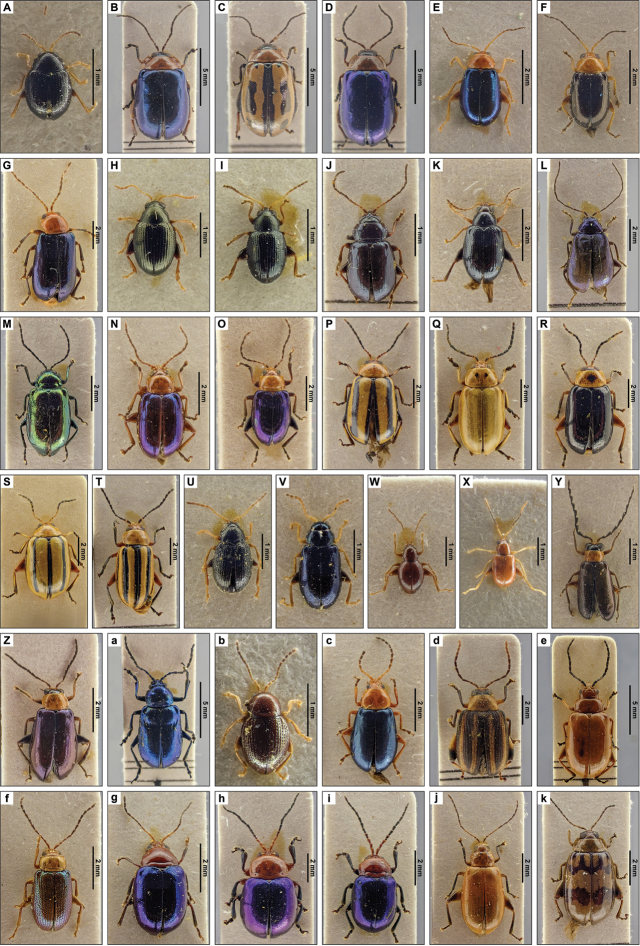
Dorsal pictures of the species of Galerucinae – Alticini from El Salvador currently present in the collections of the RBINS. Pictures of *nomina nuda* spec.s are not depicted. **A***Acallepitrixestebania***B***Alagoasaacutangula***C***A.bipunctata***D***A.ceracollis***E***Ayalaiaminor***F***A.salvadorensis***G***Cacoscelisguazapa***H***Chaetocnemafulvicornis***I***C.mexicana***J***Chalatenanganyaquadrifida***K***Cyrsylusrecticollis***L***Deuteralticalongicornis***M***Diphalticatrifiniensis***N***Diphaulacacordobae***O***D.wagneri***P***Disonychabrevilineata***Q***D.figurata***R***D.nigrita***S***D.ovata***T***D.recticollis***U***Epitrixhirtula***V***Genaphthonatransversicollis***W***Longitarsusberryi***X***L.varicornis***Y***Lupraeafulvicollis***Z***L.portilloi***a***Macrohalticasalvadorensis***b***Oreinoderaaptera***c***Phrynocephalaevicollis***d***Physimerusfemoralis***e***Platiprosopusacutangulus***f***Plectotetrasurquia***g***Syphreabalneria***h***S.pretiosa***i***S.quintanillai***j***Systenavariabilis***k***Walterianellavenustula*. High resolution images can be found at http://collections.naturalsciences.be/ssh-entomology.

**3. *Acallepitrixestebania* Bechyné & Bechyné, 1963 (Fig. 5A)**

**Published records.** AHUACHAPÁN: Apaneca, 14–17/VIII/1959 (Bechyné and Bechyné 1963). LA LIBERTAD: Hacienda Argentina, 4/IV/1960 (Bechyné and Bechyné 1963). LA PAZ: Volcan San Vicente, Finca La Paz, 1–10/VI/1959 (Bechyné and Bechyné 1963). SAN SALVADOR: Capital, 16/VI/1959 (Bechyné and Bechyné 1963), Capital, 20/VIII/1959 (Bechyné and Bechyné 1963); Cerro San Jacinto, 17/IX/1959 (Bechyné and Bechyné 1963). SANTA ANA: Volcan San Diego, 23/VI/1959 (Bechyné and Bechyné 1963).

**Specimens examined.** LA LIBERTAD: Hacienda Argentina, 4/IV/1960, Leg. J. Bechyné, Det. J. Bechyné, Paratype, 5 spec. (RBINS).

**4. *Acallepitrixhylophila* Bechyné & Bechyné, 1963**

**Published records.** LA PAZ: Volcan San Vicente, Finca La Paz, 1–10/VIII/1959 (Bechyné and Bechyné 1963).

**5. *Acallepitrixiris* Bechyné & Bechyné, 1963**

**Published records.** MORAZÁN: Perquín, 22/IX/1959 (Bechyné and Bechyné 1963). SAN SALVADOR: Cerro San Jacinto, 17/IX/1959 (Bechyné and Bechyné 1963).

**6. *Acallepitrixmorazanica* Bechyné & Bechyné, 1963**

**Published records.** MORAZÁN: Perquín, 22/IX/1959 (Bechyné and Bechyné 1963). SAN VICENTE: Volcan San Vicente, 1–10/VIII/1959 (Bechyné and Bechyné 1963).

**7. *Acallepitrixorbitalis* Bechyné & Bechyné, 1963**

**Published records.** SAN SALVADOR: Cerro San Jacinto, 17/IX/1959 (Bechyné and Bechyné 1963). SAN VICENTE: Volcan San Vicente, 1–10/VIII/1959 (Bechyné and Bechyné 1963).

**8. *Acallepitrixpersuavis* Bechyné & Bechyné, 1963**

**Published records.** AHUACHAPÁN: Apaneca, 14–17/VII/1959 (Bechyné and Bechyné 1963).

**9. *Acallepitrixponderosa* Bechyné & Bechyné, 1963**

**Published records.** LA LIBERTAD: Hacienda Chanmico, 16/VI/1959 (Bechyné and Bechyné 1963). LA PAZ: Volcan San Vicente, Finca La Paz, 1–10/VIII/1959 (Bechyné and Bechyné 1963). SAN SALVADOR: Capital, 9/VI/1959 (Bechyné and Bechyné 1963). SANTA ANA: Volcan San Diego, 22–24/VI/1959 (Bechyné and Bechyné 1963).

**10. *Acallepitrixrubrifrons* Bechyné & Bechyné, 1963**

**Published records.** LA PAZ: Volcan San Vicente, Finca La Paz, 1–10/VIII/1959 (Bechyné and Bechyné 1963). SAN SALVADOR: Capital, 11/VI/1959 (Bechyné and Bechyné 1963).

**11. *Acanthonychajacobyi* Bechyné, 1959**

**Published records.** SAN SALVADOR: 11/VI/1959, 30/VI/1959, 6/VII/1959 (Bechyné and Bechyné 1960).

**12. *Acrocyuminterposita* (Bechyné & Bechyné, 1963)**

**Published records.** AHUACHAPÁN: Apaneca, 14–17/VII/1959 (Bechyné and Bechyné 1963). SAN SALVADOR: Capital, 12/VI/1959 (Bechyné and Bechyné 1963).

**13. *Alagoasaacutangula* (Jacoby, 1886) (Fig. 5B)**

**Published records.** CHALATENANGO: La Palma, 7/VII/1959 (Bechyné and Bechyné 1959). LA LIBERTAD: Comasagua, 1/VII/1959 and 3/VII/1959 (Bechyné and Bechyné 1963). LA PAZ: Volcan San Vicente, Finca La Paz, 1–10/VIII/1959 (Bechyné and Bechyné 1963). SAN SALVADOR: Capital, 20/VI/1959 and 2/VII/1959 (Bechyné and Bechyné 1963). SANTA ANA: Volcan San Diego, 24/VI/1959 (Bechyné and Bechyné 1963).

**Specimens examined.** SAN SALVADOR: 13/VI/1959, Leg. J. Bechyné, Det. J. Bechyné, 10 spec. (RBINS).

**Remarks.**Berry (1959) also mentions this species as being present in El Salvador, but does not give specific records.

**14. *Alagoasabipunctata* (Chevrolat, 1834) (Fig. 5C)**

**Published records.** CHALATENANGO: La Palma, 7/VII/1959 (Bechyné and Bechyné 1963). CUSCATLÁN: Hacienda Colima, 27/VII,1959, (Bechyné and Bechyné 1963). LA PAZ: Volcan San Vicente, Finca La Paz, 1–10/VIII/1959 (Bechyné and Bechyné 1963). SAN SALVADOR: Capital, 8/VI/1959, 13/VI/1959, 20/VI/1959, 6/VII/1959, 20/VII/1959, 24/VII/1959, 11/VIII/1959, 23/V/1960, (Bechyné and Bechyné 1963); Lago Ilopango, 24/VII/1959 (Bechyné and Bechyné 1963); Hacienda Argentina, 17/VI/1959 and 20/VII/1959 (Bechyné and Bechyné 1963). SANTA ANA: Volcan San Diego, 22–24/VI/1959 (Bechyné and Bechyné 1963).

**Specimens examined.** SAN SALVADOR: 23/V/1960, Leg. J. Bechyné, Det. J. Bechyné, 20 spec. (RBINS).

**15. *Alagoasaceracollis* (Say, 1835) (Fig. 5D)**

**Published records.** CHALATENANGO: La Palma, 7/VII/1959 (Bechyné and Bechyné 1963). LA PAZ: Volcan San Vicente, Finca La Paz, 1–10/VIII/1959 (Bechyné and Bechyné 1963). LA LIBERTAD: Los Chorros, 27/VI/1959 (Bechyné and Bechyné 1963); Comasagua, 1/VII/1959 and 3/VII/1959 (Bechyné and Bechyné 1963); Hacienda Argentina, 17/VI/1959 (Bechyné and Bechyné 1963). MORAZÁN: Perquín, 22/IX/1959 (Bechyné and Bechyné 1963). SAN SALVADOR: 10/VI/1951, 21/VI/1951, 23/VI/1951, 13/VIII/1951, 15/IX/1951 (Bechyné 1954); Capital, 7/VI/1959, 20/VI/1959, 30/VI/1959, 28/VII/1959, 24/I/1960, 7/VI/1959, 23/V/1960 (Bechyné and Bechyné 1963). SANTA ANA: Volcan San Diego, 23/VI/1959 (Bechyné and Bechyné 1963).

**Specimens examined.** SAN SALVADOR: 23/V/1960, Leg. J. Bechyné, Det. J. Bechyné, 18 spec. (RBINS).

**16. *Alagoasadecemguttata* (Fabricius, 1801)**

**Published records.** CUSCATLÁN: El Rosario (Berry 1957). SONSONATE: San Julián (Berry 1957).

**17. *Alagoasaextrema* (Harold, 1880)**

**Published records.** LA PAZ: Volcan San Vicente, Finca La Paz, 1–10/VIII/1959 (Bechyné and Bechyné 1963). SAN SALVADOR: Capital, 24/IV/1960 (Bechyné and Bechyné 1963); Lago Ilopango, 24/VII/1959 (Bechyné and Bechyné 1963).

**18. *Alagoasaparaphana* Bechyné & Bechyné, 1963**

**Published records.** LA LIBERTAD: Comasagua, 1/VII/1959 (Bechyné and Bechyné 1963). LA PAZ: Volcan San Vicente, Finca La Paz, 1–10/VIII/1959 (Bechyné and Bechyné 1963).

**19. *Alagoasaseriata* (Baly, 1878)**

**Published records.** CHALATENANGO: La Palma, 7–8/VII/1959 (Bechyné and Bechyné 1963). CUSCATLÁN: El Rosario (Berry 1957). LA PAZ: Volcan San Vicente, Finca La Paz, 1–10/VIII/1959 (Bechyné and Bechyné 1963). SAN SALVADOR: Capital, 20/VI/1959, 30/VI/1959, 6/VII/1959 (Bechyné and Bechyné 1963). SANTA ANA: Volcan San Diego, 22/VI/1959 (Bechyné and Bechyné 1963).

**Remarks.**Berry (1959) also mentions this species as being present in El Salvador, but does not give specific records.

**20. *Alagoasavirgata* (Harold, 1880)**

**Published records.** CUSCATLÁN: El Rosario (Berry 1957).

**21. *Allochromacoccineum* Clark, 1860**

**Remarks.**Berry (1959) mentions this species as being present in El Salvador, but does not give specific records.

**22. *Alticabrevis* (Harold, 1875)**

**Published records.** AHUACHAPÁN: Hacienda Monte Cristo, 2200 m, 4–8/VI/1951 (Bechyné 1954).

**23. *Asphaeraabdominalis* (Chevrolat, 1835)**

**Published records.** AHUACHAPÁN: Apaneca, 14–17/VII/1959 (Bechyné and Bechyné 1963).

**24. *Asphaerareichei* (Harold, 1876)**

**Published records.** LA PAZ: Volcan San Vicente. Finca La Paz, 1–10/VIII/1959 (Bechyné and Bechyné 1963). SAN SALVADOR: El Salvador, IX/1950, Mertens (Bechyné 1954). SANTA ANA: Cerro Verde, 16/V/1960 (Bechyné and Bechyné 1963).

**25. *Ayalaiaminor* Bechyné & Bechyné, 1960 (Fig. 5E)**

**Published records.** AHUACHAPÁN: Apaneca, 14–17/VI/1959 (Bechyné and Bechyné 1960). LA LIBERTAD: Hacienda Argentina, 20/VII/1959 (Bechyné and Bechyné 1960). LA PAZ: Volcan San Vicente, Finca La Paz, 1–10/VIII/1959, (Bechyné and Bechyné 1960). MORAZÁN: Perquín, 22/IX/1959 (Bechyné and Bechyné 1960). SAN SALVADOR: Capital, 8/VI/1959, 19/VI/1959, 20/VII/1959, 20/VIII/1959 (Bechyné and Bechyné 1960); Cerro San Jacinto, 17/IX/1959 (Bechyné and Bechyné 1960); El Boquerón, 10/VI/1959 (Bechyné and Bechyné 1960).

**Specimens examined.** LA PAZ: Volcan San Vicente, Finca La Paz, 1/VIII/1959, Leg. J. Bechyné, Det. J. Bechyné, Paratype, 19 spec. (RBINS).

**26. *Ayalaiasalvadorensis* Bechyné & Bechyné, 1960 (Fig. 5F)**

**Published records.** CHALATENANGO: La Palma, 9/VII/1959 (Bechyné and Bechyné 1960). LA LIBERTAD: Hacienda Argentina, 17/VI/1959, 20/VII/1959, 16/V/1960 (Bechyné and Bechyné 1960). SAN SALVADOR: Capital, 19–20/VI/1959 (Bechyné and Bechyné 1960); El Boquerón, 10/VI/1959 (Bechyné and Bechyné 1960). SANTA ANA: Volcan San Diego, 22–24/VI/1959, (Bechyné and Bechyné 1960).

**Specimens examined.** LA LIBERTAD: Hacienda Argentina, 16/V/1960, Leg. J. Bechyné, Det. J. Bechyné, Paratype, 51 spec. (RBINS).

**27. *Blepharidagodmani* Jacoby 1885**

**Published records.** LA LIBERTAD: Hacienda Argentina, 19/V/1960, J. & B. Bechyné (Furth 1998).

**28. *Blepharidasuturalis* Jacoby, 1885**

**Published records.** LA LIBERTAD: Hacienda Argentina, 19/V/1960, (Furth 1998). SAN SALVADOR: El Salvador, A. Martinez Cuestas (Furth 1998); San Salvador, 30/IV–5/V/1951 (Bechyné 1954); Capital, 3/V/1960, 16/V/1960, 18/V/1960, (Bechyné and Bechyné 1963); Guazapa, 11/V/1960 (Bechyné and Bechyné 1963), 9–19/V/1958, O. L. Cartwright, at light (Furth 1998). SANTA ANA: Volcan San Diego, 22/VI/1959 (Bechyné and Bechyné 1963).

**29. *Cacoscelisguazapa* Bechyné & Bechyné, 1960 (Fig. 5G)**

**Published records.** SAN SALVADOR: Guazapa, 25/IV/1960 (Bechyné and Bechyné 1960).

**Specimens examined.** SAN SALVADOR: Guazapa, 25/IV/1960, Leg. J. Bechyné, Det. J. Bechyné, 57 spec. (RBINS).

**30. *Capraitamaculata* (Harold, 1876)**

**Published records.** AHUACHAPÁN: Apaneca, 14–17/VII/1959 (Bechyné and Bechyné 1963). SANTA ANA: Cerro Verde, 16/V/1960, (Bechyné and Bechyné 1963).

**31. *Centralaphthonadesmodita* Bechyné & Bechyné, 1960**

**Published records.** CHALATENANGO: La Palma, 7/VII/1959 (Bechyné and Bechyné 1960). MORAZÁN: Perquín, 22/IX/1959 (Bechyné and Bechyné 1960). SAN SALVADOR: Capital, 15/VI/1959, 27/VI/1959, 20/VII/1959, 20/VIII1959 (Bechyné and Bechyné 1960).

**32. *Centralaphthonadeyrollei* (Baly, 1877)**

**Published records.** SAN SALVADOR: El Boquerón, 10/VI/1959 (Bechyné and Bechyné 1960).

**33. *Centralaphthonadiversa* (Baly, 1877)**

**Published records.** CHALATENANGO: La Palma, 9/VII/1959 (Bechyné and Bechyné 1960). MORAZÁN: Perquín, 22/IX/1959 (Bechyné and Bechyné 1960). SAN SALVADOR, 11/VI/1959 (Bechyné and Bechyné 1960). SANTA ANA: Volcan San Diego, 23–24/VI/1959 (Bechyné and Bechyné 1960).

**34. *Centralaphthonadurri* Bechyné & Bechyné, 1960**

**Published records.** LA LIBERTAD: Hacienda Argentina, 17/VI/1959 (Bechyné and Bechyné 1960). SAN SALVADOR: 13/VI/1959 and 19/VI/1959 (Bechyné and Bechyné 1960).

**35. *Centralaphthonagaetana* Bechyné & Bechyné, 1960**

**Published records.** SAN SALVADOR: 3/X/1959 (Bechyné and Bechyné 1960).

**36. *Centralaphthonalessmanni* Bechyné & Bechyné, 1960**

**Published records.** LA PAZ: Volcan San Vicente, Finca La Paz, 1–10/VIII/1959 (Bechyné and Bechyné 1960). SAN SALVADOR: 19/VI/1959 (Bechyné and Bechyné 1960).

**37. *Centralaphthonanemorivaga* Bechyné & Bechyné, 1960**

**Published records.** SAN SALVADOR: Cerro San Jacinto, 17/IX/1959 (Bechyné and Bechyné 1960).

**38. *Centralaphthonaobscuripennis* (Jacoby, 1885)**

**Published records.** SAN SALVADOR: 7/VI/1959 (Bechyné and Bechyné 1960).

**39. *Centralaphthonaorbitifera* Bechyné & Bechyné, 1960**

**Published records.** LA PAZ: Volcan San Vicente, Finca La Paz, 1–10/VIII/1959 (Bechyné and Bechyné 1960). SANTA ANA: Volcan San Diego, 24/VI/1959 (Bechyné and Bechyné 1960).

**40. *Centralaphthonaperipherica* Bechyné & Bechyné, 1960**

**Published records.** MORAZÁN: Perquín, 22/IV/1959, (Bechyné and Bechyné 1960). SAN SALVADOR: 20/VIII/1959 (Bechyné and Bechyné 1960). SANTA ANA: Trifinio, 11/X/1959 (Bechyné and Bechyné 1960).

**41. *Centralaphthonaperpetualis* Bechyné & Bechyné, 1960**

**Published records.** LA LIBERTAD: Hacienda Argentina, 17/VI/1959 (Bechyné and Bechyné 1960).

**42. *Centralaphthonaprimordialis* Bechyné & Bechyné, 1960**

**Published records.** AHUACHAPÁN: Apaneca, 14–17/VII/1959 (Bechyné and Bechyné 1960).

**43. *Centralaphthonaselecta* Bechyné & Bechyné, 1960**

**Published records.** SANTA ANA: Volcan San Diego, 23–29/VI/1959 (Bechyné and Bechyné 1960).

**44. *Centralaphthonaxanthochrysa* Bechyné & Bechyné, 1960**

**Published records.** SANTA ANA: Volcan San Diego, 29/VI/1959 (Bechyné and Bechyné 1960).

**45. *Chaetocnemaacrolabris* Bechyné & Bechyné, 1963**

**Published records.** SANTA ANA: Trifinio, 11/X/1959 and 13/X/1959 (Bechyné and Bechyné 1963).

**46. *Chaetocnemaarcifera* Bechyné & Bechyné, 1963**

**Published records.** LA PAZ: Volcan San Vicente, Finca La Paz, 1–10/VIII/1959 (Bechyné and Bechyné 1963). SAN SALVADOR: Capital, 11/VI/1959 (Bechyné and Bechyné 1963); Guazapa, 10/IX/1959 (Bechyné and Bechyné 1963).

**47. *Chaetocnemabellorhina* Bechyné & Bechyné, 1963**

**Published records.** AHUACHAPÁN: Apaneca, 14–17/VII/1959 (Bechyné and Bechyné 1963).

**48. *Chaetocnemaconfinis* Crotch, 1873**

**Published records.**King and Saunders (1984) mention this species being present in El Salvador, but do not state specific records.

**49. *Chaetocnemadiegoana* Bechyné & Bechyné, 1963**

**Published records.** CHALATENANGO: La Palma, 7/VII/1959 (Bechyné and Bechyné 1963). SANTA ANA: Volcan San Diego, 23–24/VI/1959 (Bechyné and Bechyné 1963).

**50. *Chaetocnemafulvicornis* Jacoby, 1885 new record (Fig. 5H)**

**Specimens examined.** SAN SALVADOR: El Boquerón, 9/V/1960, Leg. J. Bechyné, Det. J. Bechyné, 66 spec. (RBINS).

**51. *Chaetocnemaguija* Bechyné & Bechyné, 1963**

**Published records.** SANTA ANA: Volcan San Diego, 22/VI/1959 (Bechyné and Bechyné 1963). LA PAZ: Volcan San Vicente, Finca La Paz, 1–10/VIII/1959 (Bechyné and Bechyné 1963).

**52. *Chaetocnemaitica* Bechyné & Bechyné, 1963**

**Published records.** SAN SALVADOR: Capital, 6/VIII/1959 (Bechyné and Bechyné 1963).

**53. *Chaetocnemajacinta* Bechyné & Bechyné, 1963**

**Published records.** SAN SALVADOR: Cerro San Jacinto, 17/XI/1959 (Bechyné and Bechyné 1963).

**54. *Chaetocnemalagunaria* Bechyné & Bechyné, 1963**

**Published records.** AHUACHAPÁN: Apaneca, 14–17/VII/1959 (Bechyné and Bechyné 1963).

**55. *Chaetocnemaleptocephala* Bechyné & Bechyné, 1963**

**Published records.** AHUACHAPÁN: Apaneca, 14–17/VII/1959 (Bechyné and Bechyné 1963).

**56. *Chaetocnemamexicana* Baly, 1877 (Fig. 5I)**

**Published records.** CHALATENANGO: La Palma, 7/VII/1959 (Bechyné and Bechyné 1963). LA LIBERTAD: Hacienda Chanmico, 3/IX/1959 (Bechyné and Bechyné 1963); Hacienda Argentina, 20/VII/1959 (Bechyné and Bechyné 1963). SAN SALVADOR: Guazapa, 11/V/1960 (Bechyné and Bechyné 1963). SANTA ANA: Volcan Diego, 22–23/VI/1959 (Bechyné and Bechyné 1963); Trifinio, 14/X/1959 (Bechyné and Bechyné 1963).

**Specimens examined.** SAN SALVADOR: Guazapa, 11/V/1960, Leg. J. Bechyné, Det. J. Bechyné, 102 spec. (RBINS).

**57. *Chaetocnemanepotica* Bechyné & Bechyné, 1963**

**Published records.** MORAZÁN: Perquín, 23/IX/1959 (Bechyné and Bechyné 1963).

**58. *Chaetocnemaobtusilabris* Bechyné & Bechyné, 1963**

**Published records.** MORAZÁN: Perquín, 22/IX/1959 (Bechyné and Bechyné 1963). SANTA ANA: Volcan San Diego, 22/VI/1959 (Bechyné and Bechyné 1963).

**59. *Chaetocnemaperquinensis* Bechyné & Bechyné, 1963**

**Published records.** MORAZÁN: Perquín, 22/IX/1959 (Bechyné and Bechyné 1963). SAN SALVADOR: Capital, 19/VI/1959, 28/VII/1959, 27/IX/1959 (Bechyné and Bechyné 1963); El Boquerón, 20/VIII/1959 (Bechyné and Bechyné 1963). SANTA ANA: Trifinio, 14/X/1959 (Bechyné and Bechyné 1963).

**60. *Chaetocnemasitarina* Bechyné & Bechyné, 1963**

**Published records.** AHUACHAPÁN: Apaneca, 14–17/VII/1959 (Bechyné and Bechyné 1963).

**61. *Chaetocnemavega* Bechyné & Bechyné, 1963**

**Published records.** LA PAZ: Volcan San Vicente, Finca La Paz, 1–10/VIII/1959 (Bechyné and Bechyné 1963). SANTA ANA: Volcan San Diego, 22/VI/1959 (Bechyné and Bechyné 1963). SAN SALVADOR: Cerro San Jacinto, 17/IX/1959 (Bechyné and Bechyné 1963).

**62. *Chalatenanganyaquadrifida* Bechyné & Bechyné, 1963 (Fig. 5J)**

**Published records.** CHALATENANGO: La Palma, 9/VII/1959 (Bechyné and Bechyné 1963). LA LIBERTAD: Comasagua, 1/VII/1959 and 3/VII/1959 (Bechyné and Bechyné 1963); Los Chorros, 29/VI/1959 (Bechyné and Bechyné 1963); Hacienda Argentina, 20/VII/1959 and 18/V/1960 (Bechyné and Bechyné 1963). LA UNIÓN: Cutuco, 2–4/VI/1959 (Bechyné and Bechyné 1963). SAN SALVADOR: Capital, 12/VI/1959, 16/VI/1959, 30/VI/1959 (Bechyné and Bechyné 1963); El Boquerón, 10/VI/1959 (Bechyné and Bechyné 1963); Cerro San Jacinto, 17/IX/1959 (Bechyné and Bechyné 1963). SANTA ANA: Volcan San Diego, 23–24/VI/1959 (Bechyné and Bechyné 1963).

**Specimens examined.** LA LIBERTAD: Hacienda Argentina, 18/V/1960, Leg. J. Bechyné, Det. J. Bechyné, Paratype, 53 spec. (RBINS).

**63. *Cyrsylusrecticollis* Jacoby, 1891 (Fig. 5K)**

**Published records.** LA LIBERTAD: Comasagua, 1/VII/1959 and 3/VII/1959 (Bechyné and Bechyné 1963). MORAZÁN: Perquín, 25/IX/1959 (Bechyné and Bechyné 1963). SAN SALVADOR: Capital, 6/VI/1959, 11/VI/1959, 18–19/VI/1959, 21/VII/1959, 27/IX/1959, (Bechyné and Bechyné 1963); Cerro San Jacinto, 17/IX/1959 (Bechyné and Bechyné 1963); Guazapa, 10/IV/1960 (Bechyné and Bechyné 1963). SANTA ANA: Volcan San Diego, 24/VI/1959 (Bechyné and Bechyné 1963). USULUTÁN: Berlín, V/1898, A. & F. Solari (Bechyné 1957).

**Specimens examined.** LA LIBERTAD: Santa Tecla, 28/IV/1960, Leg. J. Bechyné, Det. J. Bechyné, 24 spec. (RBINS); Tamanigue, 4/V/1960, Leg. J. Bechyné, Det. J. Bechyné, 25 spec. (RBINS).

**64. *Deuteralticalongicornis* (Jacoby, 1891) (Fig. 5L)**

**Published records.** AHUACHAPÁN: Apaneca, 14–17/VII/1959 (Bechyné and Bechyné 1960).SAN SALVADOR: Capital, 8/VI/1959 (Bechyné and Bechyné 1960); El Boquerón, 10/VI/1959 (Bechyné and Bechyné 1960).

**Specimens examined.** SAN SALVADOR: El Boquerón, 9/V/1960, Leg. J. Bechyné, Det. J. Bechyné, 53 spec. (RBINS).

**Remarks.**Berry (1959) also mentions this species as being present in El Salvador, but does not give specific records.

**65. *Dinalticaanilina* Bechyné & Bechyné, 1963**

**Published records.** MORAZÁN: Perquín, 22/IX/1959 (Bechyné and Bechyné 1963).

**66. *Diphalticatrifiniensis* Bechyné & Bechyné, 1960 (Fig. 5M)**

**Published records.** SANTA ANA: Trifinio, 11/X/1959 and 6–8/III/1960 (Bechyné and Bechyné 1960).

**Specimens examined.** SANTA ANA: Trifinio, 6/III/1960, Leg. J. Bechyné, Det. J. Bechyné, Paratype, 51 spec. (RBINS).

**67. *Diphaulacaaulicaaulica* Barber, 1941**

**Published records.** SAN SALVADOR, 5/VII/1951 (Bechyné 1954).

**Specimens examined.** UNKNOWN PROVINCE: Cristobal, 20/V/1959, Leg. J. Bechyné, Det. J. Bechyné, 13 spec. (RBINS).

**Remarks.** The RBINS spec. are labeled as *D.panama*, which is currently a junior synonym of *D.aulicaaulica* (Olivier, 1808); see Furth and Savini (1996). The record from Bechyné (1954) names this species as *Diphaulacaaulica* (Olivier, 1888).

**68. *Diphaulacaaulicacordobae* Barber, 1941 (Fig. 5N)**

**Published records.** AHUACHAPÁN: Apaneca, 14–17/VII/1959 (Bechyné and Bechyné 1960). CUSCATLÁN: Hacienda Colima, 22/VII/1959 (Bechyné and Bechyné 1960). LA LIBERTAD: Hacienda Chanmico, 3/IX/1959 and, 20/VI/1960 (Bechyné and Bechyné 1960); Hacienda Argentina, 15/VI/1959, and 20/VII/1959 (Bechyné and Bechyné 1960); Los Chorros, 29/VI/1959 (Bechyné and Bechyné 1960); Comasagua, 1/VII/1959 and 3/VII/1959 (Bechyné and Bechyné 1960). LA PAZ: Volcan San Vicente, Finca La Paz, 1–10/VIII/1959 (Bechyné and Bechyné 1960). LA UNIÓN: Cutuco, 2–4/VI/1959 (Bechyné and Bechyné 1960). SAN SALVADOR: Capital, 7/VI/1959, 11/VI/1959, 13/VI/1959, 15/VI/1959, 19/VI/1959, 20/VI/1959, 20/VII/1959, 24/VII/1959, 28/VII/1959, 20/VIII/1959, 24/IV/1960 (Bechyné and Bechyné 1960); Cerro San Jacinto, 17/IX/1959 (Bechyné and Bechyné 1960); Lago Ilopango, 24/VII/1959 (Bechyné and Bechyné 1960); El Boquerón, 10/VI/1959 and 20/VIII/1959 (Bechyné and Bechyné 1960); Guazapa, 10/IX/1959 and 25/IV/1960 (Bechyné and Bechyné 1960). SANTA ANA: Volcan San Diego, 22–24/VI/1959 (Bechyné and Bechyné 1960).

**Specimens examined.** LA LIBERTAD: Comasagua, 1/VII/1959, Leg. J. Bechyné, Det. J. Bechyné, 31 spec. (RBINS). LA UNIÓN: Cutuco, 3/VI/1959, Leg. J. Bechyné, Det. J. Bechyné, 24 spec. (RBINS).

**69. *Diphaulacasalvadorensis* Bechyné & Bechyné, 1960**

**Published records.** CHALATENANGO: La Palma, 7/VII/1959 (Bechyné and Bechyné 1960).LA LIBERTAD: Los Chorros, 29/VI/1959 (Bechyné and Bechyné 1960). SAN SALVADOR, 7/VI/1959, 15/VI/1959, 12/VII/1959, 21/VII/1959 (Bechyné and Bechyné 1960).

**70. *Diphaulacawagneri* Harold, 1875 (Fig. 5O)**

**Published records.** AHUACHAPÁN, Apaneca, 14–17/VII/1959 (Bechyné and Bechyné 1960). CHALATENANGO: La Palma, 9/VII/1959 (Bechyné and Bechyné 1960).LA LIBERTAD: Comasagua, 1/VII/1959 and 3/VII/1959 (Bechyné and Bechyné 1960).SAN SALVADOR: Cerro San Jacinto, 17/IX/1959 (Bechyné and Bechyné 1960); El Boquerón, 10/VI/1959 and 20/VIII/1959 (Bechyné and Bechyné 1960).

**Specimens examined.** CHALATENANGO: La Palma, 9/VII/1959, Leg. J. Bechyné, Det. J. Bechyné, 11 spec..

**71. *Disonychabrevilineata* Jacoby, 1884 (Fig. 5P)**

**Published records.** CHALATENANGO: La Palma, 7/VII/1959 (Bechyné and Bechyné 1963). LA LIBERTAD: Hacienda Chanmico, 20/VI/1960 (Bechyné and Bechyné 1963); Comasagua, 3/VII/1959 (Bechyné and Bechyné 1963). LA PAZ: Volcan San Vicente, Finca La Paz, 1–10/VIII/1959 (Bechyné and Bechyné 1963). SAN SALVADOR: Capital, 30/VI/1959, 6–7/VII/1959, 28/VII/1959, 23/V/1960 (Bechyné and Bechyné 1963); Lago Ilopango, 24/VII/1959 (Bechyné and Bechyné 1963). SANTA ANA: Volcan San Diego, 23–24/VI/1959 (Bechyné and Bechyné 1963).

**Specimens examined.** LA LIBERTAD: Hacienda Chanmico, 20/VI/1960, Leg. J. Bechyné, Det. J. Bechyné, 10 spec. (RBINS); Santa Tecla, 28/IV/1960, Leg. J. Bechyné, Det. J. Bechyné, 25 spec. (RBINS); Tamanigue, 4/V/1960, Leg. J. Bechyné, Det. J. Bechyné, 5 spec. (RBINS). SAN SALVADOR: 23/V/1960, Leg. J. Bechyné, Det. J. Bechyné, 8 spec. (RBINS). SANTA ANA: Volcan San Diego, 23/VI/1959, Leg. J. Bechyné, Det. J. Bechyné, 13 spec. (RBINS).

**Remarks.**Berry (1959) also mentions this species as being present in El Salvador, but does not give specific records.

**72. *Disonychabrunneofasciata* Jacoby, 1884**

**Published records.** LA PAZ: Volcan San Vicente, Finca La Paz, 1–10/VIII/1959 (Bechyné and Bechyné 1963).

**73. *Disonychacollata* (Fabricius, 1801)**

**Published records.** SAN SALVADOR: Capital, 24/IV/1960 (Bechyné and Bechyné 1963).

**74. *Disonychadorsata* Harold, 1880**

**Published records.** CUSCATLÁN: El Rosario (Berry 1957).

**75. *Disonychafigurata* Jacoby, 1884 (Fig. 5Q)**

**Published records.** SANTA ANA: Hacienda Los Planes, 1800 m, Metapan Mts., 24–25/VIII/1951 (Bechyné 1954).

**Specimens examined.** SAN SALVADOR: 19/VI/1960, Leg. J. Bechyné, Det. J. Bechyné, 39 spec. (RBINS).

**Remarks.**Berry (1959) also mentions this species as being present in El Salvador, but does not give specific records.

**76. *Disonychafumatalabiata* Jacoby, 1901**

**Published records.** AHUACHAPÁN: Apaneca, 14–17/VII/1959 (Bechyné and Bechyné 1963). SAN SALVADOR: El Boquerón, 10/VI/1959 and 20/VIII/1959 (Bechyné and Bechyné 1963).

**77. *Disonychaglabrata* (Fabricius, 1781)**

**Published records.** LA LIBERTAD: Los Chorros, 26/VI/1959, (Bechyné & Bechyné, 1963); Comasagua, 3/VII/1959, (Bechyné & Bechyné, 1963); Santa Tecla (Berry 1957). SANTA ANA: Volcan San Diego, 22–29/VI/1959 (Bechyné and Bechyné 1963). LA PAZ: Volcan San Vicente, Finca La Paz, 1–10/VIII/1959 (Bechyné and Bechyné 1963). SAN SALVADOR: Capital, 12/VI/1959, 15/VI/1959, 27/VI/1959, 30/VI/1959 (Bechyné and Bechyné 1963).

**78. *Disonychaguatemalensis* Jacoby, 1884**

**Published records.** CUSCATLÁN: Hacienda Colima, 22/VII/1959 (Bechyné and Bechyné 1963).

**79. *Disonychamexicana* Jacoby, 1884**

**Published records.** CHALATENANGO: La Palma, 7/VII/1959, (Bechyné and Bechyné 1963). LA LIBERTAD: Comasagua, 3/VII/1959 (Bechyné and Bechyné 1963). MORAZÁN: Perquín, 22/IX/1959 (Bechyné and Bechyné 1963). SAN SALVADOR: Lago Ilopango, 24/VII/1959 (Bechyné and Bechyné 1963); Guazapa, 10/IX/1959 (Bechyné and Bechyné 1963).

**80. *Disonychamilitaris* Jacoby, 1884**

**Published records.** LA PAZ: Volcan San Vicente, Finca La Paz, 1–10/VIII/1959 (Bechyné and Bechyné 1963). SAN SALVADOR, 13/VIII/1951 (Bechyné 1954).

**Remarks.**Berry (1959) also mentions this species as being present in El Salvador, but does not give specific records.

**81. *Disonychanigrita* Jacoby, 1884 (Fig. 5R)**

**Published records.** CUSCATLÁN: Hacienda Colima, 22/VII/1959 (Bechyné and Bechyné 1963). LA LIBERTAD: Comasagua, 1/VII/1959 and 3/VII/1959 (Bechyné and Bechyné 1963); Hacienda Chanmico, 3/IX/1959 and, 20/VI/1960 (Bechyné and Bechyné 1963). SAN SALVADOR: Capital, 6/VII/1959 and 28/VII/1959 (Bechyné and Bechyné 1963); Lago Ilopango, 29/VII/1959 (Bechyné and Bechyné 1963). SANTA ANA: Volcan San Diego, 23/VI/1959 (Bechyné and Bechyné 1963).

**Specimens examined.** LA LIBERTAD: Hacienda Chanmico, 20/VI/1960, Leg. J. Bechyné, Det. J. Bechyné, 8 spec. (RBINS).

**Remarks.**Berry (1959) also mentions this species as being present in El Salvador, but does not give specific records.

**82. *Disonychaovata* Blake, 1931 (Fig. 5S)**

**Published records.** AHUACHAPÁN: Los Ausoles, 26/I/1960 (Bechyné and Bechyné 1963). CHALATENANGO: La Palma, 7–8/VII/1959 (Bechyné and Bechyné 1963). LA LIBERTAD: Comasagua, 3/VII/1959 (Bechyné and Bechyné 1963); Santa Tecla (Berry 1957). SAN SALVADOR: Capital, 8/VI/1959, 19/VI/1959, 6/VII/1959, 20/VII/1959, 3/X/1959, 23/V/1960 (Bechyné and Bechyné 1963); El Boquerón, 10/VI/1959 (Bechyné and Bechyné 1963); Guazapa, 10/IX/1959 (Bechyné and Bechyné 1963).

**Specimens examined.** SAN SALVADOR: Cerro San Jacinto, 17/IX/1959, Leg. J. Bechyné, Det. J. Bechyné, 5 spec. (RBINS), 13/VI/1959, Leg. J. Bechyné, Det. J. Bechyné, 5 spec. (RBINS), 22/I0/1959, Leg. J. Bechyné, Det. J. Bechyné, 4 spec. (RBINS), 23/V/1960, Leg. J. Bechyné, Det. J. Bechyné, 5 spec. (RBINS).

**83. *Disonychaquinquelineata* (Latreille, 1881)**

**Published records.** AHUACHAPÁN: Apaneca, 14–17/VII/1959 (Bechyné and Bechyné 1963). CUSCATLÁN: Volcan San Vicente, Finca El Carmen, 1300 m, 11–16/VI/1951 (Bechyné 1954). SAN SALVADOR: 23/VI/1951 (Bechyné 1954).

**84. *Disonycharecticollis* (Jacoby, 1884) (Fig. 5T)**

**Published records.** AHUACHAPÁN: Apaneca, 27/I/1959 and 14–17/VII/1959 (Bechyné and Bechyné 1963). SAN SALVADOR: El Boquerón, 10/VI/1959 (Bechyné and Bechyné 1963); San Salvador (Berry 1957).

**Specimens examined.** AHUACHAPÁN: Apaneca, 15/VII/1959, Leg. J. Bechyné, Det. J. Bechyné, 24 spec. (RBINS).

**85. *Disonychascriptipennis* (Jacoby, 1884)**

**Published records.** SAN SALVADOR: Capital, 28/VII/1959 (Bechyné and Bechyné 1963).

**86. *Disonychasteinheili* Harold, 1876**

**Published records.** SAN SALVADOR: El Boquerón, 10/VI/1959 (Bechyné and Bechyné 1963).

**87. *Disonychatrifasciata* Clark, 1865**

**Published records.** LA LIBERTAD: Comasagua, 3/VII/1959 (Bechyné and Bechyné 1963). SAN SALVADOR: Capital, 12/VI/1959, 20/VII/1959, 28/VII/1959 (Bechyné and Bechyné 1963). Guazapa, 10/IX/1959 (Bechyné and Bechyné 1963). SANTA ANA: Volcan San Diego, 26/VI/1959 (Bechyné and Bechyné 1963).

**88. *Disonychavera* Bechyné & Bechyné, 1963**

**Published records.** AHUACHAPÁN: Apaneca, 14–17/VII/1959 (Bechyné and Bechyné 1963). SAN SALVADOR: Capital, 8/VI/1959 (Bechyné and Bechyné 1963).

**89. *Epitrixanahoria* Bechyné & Bechyné, 1960**

**Published records.** CHALATENANGO: La Palma, 9/VII/1959 (Bechyné and Bechyné 1960). MORAZÁN: Perquín, 22/IX/1959 (Bechyné and Bechyné 1960). SANTA ANA: Volcan San Diego, 24/VI/1959 (Bechyné and Bechyné 1960).

**90. *Epitrixangelina* Bechyné & Bechyné, 1960**

**Published records.** AHUACHAPÁN: Apaneca, 14–17/VII/1959 (Bechyné and Bechyné 1960).

**91. *Epitrixapanecana* Bechyné & Bechyné, 1960**

**Published records.** AHUACHAPÁN: Apaneca, 14–17/VII/1959 (Bechyné and Bechyné 1960). LA LIBERTAD: Comasagua, 3/VII/1959 (Bechyné and Bechyné 1960). LA PAZ: Volcan San Vicente, Finca La Paz, 1–10/VII/1959 (Bechyné and Bechyné 1960). SAN SALVADOR: El Boquerón 10/VI/1959, (Bechyné and Bechyné 1960).

**92. *Epitrixatripessilvicola* Bechyné & Bechyné, 1960**

**Published records.** SANTA ANA: Trifinio, 11–15/X/1959 (Bechyné and Bechyné 1960).

**93. *Epitrixauricoma* Bechyné & Bechyné, 1960**

**Published records.** SANTA ANA: Trifinio, 13/X/1959 (Bechyné and Bechyné 1960).

**94. *Epitrixdilaticornis* Jacoby, 1885**

**Published records.** LA PAZ: Volcan San Vicente, Finca La Paz, 1–10/VIII/1959 (Bechyné and Bechyné 1960).

**95. *Epitrixhirtula* Harold, 1875 (Fig. 5U)**

**Published records.** SAN SALVADOR: El Boquerón, 9/V/1960 (Bechyné and Bechyné 1960). SANTA ANA: Trifinio, 11–15/X/1959 (Bechyné and Bechyné 1960).

**Specimens examined.** SAN SALVADOR: El Boquerón, 9/V/1960, Leg. J. Bechyné, Det. J. Bechyné, 53 spec., (RBINS).

**96. *Epitrixintegralis* Bechyné & Bechyné, 1960**

**Published records.** AHUACHAPÁN: Apaneca, 14–17/VII/1959 (Bechyné and Bechyné 1960).

**97. *Epitrixlacustris* Bechyné & Bechyné, 1960**

**Published records.** AHUACHAPÁN: Apaneca, 14–17/VII/1959 (Bechyné and Bechyné 1960). LA PAZ: Volcan San Vicente, Finca La Paz, 1–10/VIII/1959 (Bechyné and Bechyné 1960). SAN SALVADOR: Capital, 20/VIII/1959 (Bechyné and Bechyné 1960); Cerro San Jacinto, 17/IX/1959 (Bechyné and Bechyné 1960).

**98. *Epitrixnicolina* Bechyné & Bechyné, 1960**

**Published records.** SAN SALVADOR: 13/VI/1959 and 20/VII/1959 (Bechyné and Bechyné 1960). SANTA ANA: Volcan San Diego, 23/VI/1959 (Bechyné and Bechyné 1960).

**99. *Epitrixninfa* Bechyné & Bechyné, 1960**

**Published records.** AHUACHAPÁN: Apaneca (Laguna de las ninfas), 14–17/VII/1959 (Bechyné and Bechyné 1960). LA LIBERTAD: Hacienda Argentina, 20/VII/1959 (Bechyné and Bechyné 1960).

**100. *Epitrixnycteroptera* Bechyné & Bechyné, 1960**

**Published records.** SANTA ANA: Trifinio, 11/X/1959 (Bechyné and Bechyné 1960).

**101. *Epitrixperquinensis* Bechyné & Bechyné, 1960**

**Published records.** MORAZÁN: Perquín, 22/IX/1959 (Bechyné and Bechyné 1960).

**102. *Epitrixthoracolysa* Bechyné & Bechyné, 1960**

**Published records.** SANTA ANA: Volcan San Diego, 27/VI/1959 (Bechyné and Bechyné 1960).

**103. *Epitrixtriangularis* Bechyné & Bechyné, 1960**

**Published records.** SANTA ANA: Trifinio, 11–14/X/1959 (Bechyné and Bechyné 1960).

**104. *Epitrixvincentina* Bechyné & Bechyné, 1960**

**Published records.** LA PAZ: Volcan San Vicente, Finca La Paz, 1–10/VIII/1959 (Bechyné and Bechyné 1960).

**105. *Genaphthonatransversicollis* (Jacoby, 1885) (Fig. 5V)**

**Published records.** AHUACHAPÁN: Apaneca, 14–17/VIII/1959, (Bechyné & Bechyné, 1960). CHALATENANGO: La Palma, 7/VII/1959, (Bechyné & Bechyné, 1960). LA LIBERTAD: Hacienda Argentina, 17/VI/1959, (Bechyné & Bechyné, 1960). Hacienda Argentina, 30/IX/1959, (Bechyné & Bechyné, 1960). Comasagua, 1/VII/1959, (Bechyné & Bechyné, 1960). SAN SALVADOR: Capital, 9/VI/1959, 12/VI/1959, 19/VI/1959, 31/IX/1959 (Bechyné and Bechyné 1960). El Boquerón, 10/VI/1959 and 20/VIII/1959 (Bechyné and Bechyné 1960). SANTA ANA: Volcan San Diego, 29/VI/1959, (Bechyné & Bechyné, 1960). Trifinio, 10/X/1959 (Bechyné & Bechyné, 1960).

**Specimens examined.** LA LIBERTAD: Hacienda Argentina, 18/V/1960, Leg. J. Bechyné, Det. J. Bechyné, 54 spec. (RBINS).

**106. *Genaphthonavirkkii* Bechyné & Bechyné, 1960**

**Published records.** AHUACHAPÁN: Apaneca, 14–17/VII/1959 (Bechyné and Bechyné 1960).

**107. *Heikertingerellaallopantha* Bechyné & Bechyné, 1960**

**Published records.** LA PAZ: Volcan San Vicente, Finca La Paz, 1–10/VIII/1959 (Bechyné and Bechyné 1960). SAN SALVADOR: 12/VI/1959, 20/VIII/1959, 27/IX/1959, 30/IX/1959, (Bechyné and Bechyné 1960). SANTA ANA: Volcan San Diego, 23/VI/1959 (Bechyné and Bechyné 1960).

**108. *Heikertingerellabrachycaula* Bechyné & Bechyné, 1960**

**Published records.** SANTA ANA : Trifinio, 11/X/1959 (Bechyné and Bechyné 1960).

**109. *Heikertingerellabrachygena* Bechyné & Bechyné, 1960**

**Published records.** SANTA ANA: Trifinio, 14/X/1959 (Bechyné and Bechyné 1960).

**110. *Heikertingerellahamagira* Bechyné & Bechyné, 1960**

**Published records.** MORAZÁN: Perquín, 22/IX/1959 (Bechyné and Bechyné 1960).

**111. *Heikertingerellairrahetai* Bechyné & Bechyné, 1960**

**Published records.** SANTA ANA: Volcan San Diego, 29/VI/1959 (Bechyné and Bechyné 1960).

**112. *Heikertingerellamacrogena* Bechyné & Bechyné, 1960**

**Published records.** AHUACHAPÁN: Apaneca, 14–17/?/1959 (Bechyné and Bechyné 1960). CHALATENANGO: La Palma, 7/VII/1959 (Bechyné and Bechyné 1960). MORAZÁN: Perquín, 22/IX/1959 (Bechyné and Bechyné 1960). LA PAZ: Volcan San Vicente, Finca La Paz, 1–10/VIII/1959 (Bechyné and Bechyné 1960). SAN SALVADOR: Capital, 15/VI/1959, 19/VI/1959, 20/VII/1959 (Bechyné and Bechyné 1960); Lago Ilopango, 24/VII/1959 (Bechyné and Bechyné 1960).

**113. *Heikertingerellasiliconia* Bechyné & Bechyné, 1960**

**Published records.** AHUACHAPÁN: Apaneca, 14–17/VII/1959 (Bechyné and Bechyné 1960).

**114. *Heikertingerellatrifiniensis* Bechyné & Bechyné, 1960**

**Published records.** SANTA ANA: Trifinio, 14/X/1959 (Bechyné and Bechyné 1960).

**115. *Heikertingerellavariabilis* (Jacoby, 1885)**

**Published records.** LA PAZ: Volcan San Vicente, Finca La Paz, 1–10/VIII/1959 (Bechyné and Bechyné 1960). SAN SALVADOR: 6/VII/1959 (Bechyné and Bechyné 1960).

**116. *Heikertingerellaxanthocarya* Bechyné & Bechyné, 1960**

**Published records.** MORAZÁN: Perquín, 22/IX/1959 (Bechyné and Bechyné 1960). SAN SALVADOR: Guazapa, 10/IX/1959 (Bechyné and Bechyné 1960). SANTA ANA: Trifinio, 14/X/1959 (Bechyné and Bechyné 1960).

**117. *Longitarsusargopterus* Bechyné & Bechyné, 1960**

**Published records.** LA PAZ: Volcan San Vicente, Finca La Paz, 1–10/VIII/1959 (Bechyné and Bechyné 1960).

**118. *Longitarsusasteriscus* Bechyné & Bechyné, 1960**

**Published records.** MORAZÁN: Perquín, 22/IX/1959 (Bechyné and Bechyné 1960). SAN SALVADOR: 20/VII/1959 (Bechyné and Bechyné 1960).

**119. *Longitarsusberryi* Bechyné & Bechyné, 1960 (Fig. 5W)**

**Published records.** SANTA ANA: Trifinio, 11–14/X/1959 and 8–9/III/1960 (Bechyné and Bechyné 1960).

**Specimens examined.** SANTA ANA: Trifinio, 8/III/1960, Leg. J. Bechyné, Det. J. Bechyné, Paratype, 52 spec. (RBINS).

**120. *Longitarsuscolumbicuscentroamericanus* Bechyné & Bechyné, 1960**

**Published records.** CHALATENANGO: La Palma, 7/VII/1959 (Bechyné and Bechyné 1960). LA PAZ: Volcan San Vicente, Finca La Paz, 1–10/VIII/1959 (Bechyné and Bechyné 1960). MORAZÁN: Perquín, 22/IX/1959 (Bechyné and Bechyné 1960). SAN SALVADOR, 19/VI/1959, 20/VII/1959, 27/VII/1959 (Bechyné and Bechyné 1960).

**121. *Longitarsusgerodontus* Bechyné & Bechyné, 1960**

**Published records.** MORAZÁN: Perquín, 22/IX/1959 (Bechyné and Bechyné 1960).

**122. *Longitarsusorphanus* Bechyné & Bechyné, 1960**

**Published records.** MORAZÁN: Perquín, 25/VIII/1959 (Bechyné and Bechyné 1960). SANTA ANA: Trifinio, 11/X/1959 (Bechyné and Bechyné 1960).

**123. *Longitarsusperichromus* Bechyné & Bechyné, 1960**

**Published records.** LA PAZ: Volcan San Vicente, Finca La Paz, 1–10/VIII/1959 (Bechyné and Bechyné 1960). MORAZÁN: Perquín, 28/IX/1959 (Bechyné and Bechyné 1960).

**124. *Longitarsusscurrilis* Bechyné & Bechyné, 1960**

**Published records.** LA PAZ: Volcan San Vicente, Finca La Paz, 1–10/VIII/1959 (Bechyné and Bechyné 1960).

**125. *Longitarsusseraphinus* Bechyné & Bechyné, 1960**

**Published records.** AHUACHAPÁN: Apaneca, 14–17/VII/1959 (Bechyné and Bechyné 1960).

**126. *Longitarsussomaticus* Bechyné & Bechyné, 1960**

**Published records.** SAN SALVADOR: 15/VI/1959 and 20/VIII/1959 (Bechyné and Bechyné 1960).

**127. *Longitarsussparnus* Bechyné & Bechyné, 1960**

**Published records.** AHUACHAPÁN: Apaneca, 14–17/VII/1959 (Bechyné and Bechyné 1960).

**128. *Longitarsusvaricornis* Suffrian, 1868 (Fig. 5X)**

**Published records.** SANTA ANA: Volcan San Diego, 29/VI/1959 (Bechyné and Bechyné 1960).

**Specimens examined.** LA LIBERTAD: Hacienda San Diego, 28/IV/1960, Leg. J. Bechyné, Det. J. Bechyné, 61 spec. (RBINS).

**129. *Luperalticacayetunya* (Bechyné & Bechyné, 1960)**

**Published records.** SANTA ANA: Volcan San Diego, 22–24/VI/1959 (Bechyné and Bechyné 1960); Trifinio, 10–14/X/1959 (Bechyné and Bechyné 1960).

**130. *Luperalticasylvia* (Bechyné & Bechyné, 1960)**

**Published records.** CHALATENANGO: La Palma, 7/VII/1959 (Bechyné and Bechyné 1960). LA LIBERTAD: Hacienda Argentina, 20/VII/1959 (Bechyné and Bechyné 1960); Comasagua, 3/VII/1959 (Bechyné and Bechyné 1960). SAN SALVADOR: Capital, 8/VI/1959, 11/VI/1959, 13/VI/1959, 18–19/VI/1959, 6/VII/1959, 28/VII/1959 (Bechyné and Bechyné 1960); El Boquerón, 10/VI/1956 (Bechyné and Bechyné 1960). SANTA ANA: Volcan San Diego, 29/VI/1959 (Bechyné and Bechyné 1960).

**131. *Luperalticaustulatacentralis* (Bechyné, 1955)**

**Published records.** LA LIBERTAD: Comasagua, 3/VII/1959 (Bechyné and Bechyné 1960).

**132. *Lupraeaacanthonychina* Bechyné & Bechyné, 1960**

**Published records.** CHALATENANGO: La Palma, 9/VII/1959 (Bechyné and Bechyné 1960). SANTA ANA: Volcan San Diego, 24/VI/1959 (Bechyné and Bechyné 1960).

**133. *Lupraeafulvicollis* Jacoby, 1885 (Fig. 5Y)**

**Published records.** CHALATENANGO: La Palma, 7/VII/1959 (Bechyné and Bechyné 1960). LA PAZ: Volcan San Vicente, Finca La Paz, 1–10/VIII/1959 (Bechyné and Bechyné 1960). SAN SALVADOR: Capital, 8/VI/1959, 11/VI/1959, 19/VI/1959, 21/VII/1959, 22/IX/1959, 1/I/1960 (Bechyné and Bechyné 1960); Hacienda Argentina, 20/VII/1959 (Bechyné and Bechyné 1960).

**Specimens examined.** SAN SALVADOR: 2/VII/1960, Leg. J. Bechyné, Det. J. Bechyné, 8 spec. (RBINS).

**134. *Lupraeanigricollis* (Jacoby, 1891)**

**Published records.** AHUACHAPÁN: Apaneca, 14–17/VII/1959 (Bechyné and Bechyné 1960).

**135. *Lupraeaportilloi* Bechyné & Bechyné, 1960 (Fig. 5Z)**

**Published records.** SAN SALVADOR: 7/VI/1959, 8/VI/1959, 12/VI/1959, 13/VI/1959, 19/VI/1959, 21/VII/1959, 31/V/1960, 19/VI/1960 (Bechyné and Bechyné 1960).

**Specimens examined.** SAN SALVADOR: 31/V/1960, Leg. J. Bechyné, Det. J. Bechyné, Paratype, 34 spec. (RBINS).

**136. *Lupraeasantaneca* Bechyné & Bechyné, 1960**

**Published records.** SANTA ANA: Volcan San Diego, 24/VI/1959 (Bechyné and Bechyné 1960).

**137. *Lysathiacomasagua* Bechyné & Bechyné, 1960**

**Published records.** LA LIBERTAD: Comasagua, 3/VII/1959 (Bechyné and Bechyné 1960).

**138. *Lysathiasimplex* (Jacoby, 1891)**

**Published records.** AHUACHAPÁN: Apaneca, 14–17/VII/1959 (Bechyné and Bechyné 1960). SAN SALVADOR: 24/VII/1959 and 20/VIII/1959 (Bechyné and Bechyné 1960). SANTA ANA: Volcan San Diego, 24/VI/1959 (Bechyné and Bechyné 1960).

**139. *Lysathiavolcanica* Bechyné & Bechyné, 1960**

**Published records.** AHUACHAPÁN: Apaneca, 14–17/VII/1959 (Bechyné and Bechyné 1960). SANTA ANA: Volcan San Diego, 24/VI/1959 (Bechyné and Bechyné 1960).

**140. *Macrohalticasalvadorensis* Bechyné & Bechyné, 1954 (Fig. 5a)**

**Published records.** AHUACHAPÁN: Apaneca, 14–17/VII/1959 (Bechyné and Bechyné 1960), 9/VI/1926, Salman, K.A., USNM (Santisteban 2006); Laguna de la Ninfas, 1630 m, 18/VII/1951 (Bechyné 1954). LA LIBERTAD: Comasagua, 1/VII/1959 (Bechyné and Bechyné 1960); Santa Tecla, ll/X/1956, Berry, RA., USNM (Santisteban 2006); Valiano, 5500 ft, Berry, RA., USNM (Santisteban 2006). CHALATENANGO: La Palma, 7/VII/1959 (Bechyné and Bechyné 1960). SAN SALVADOR: 18/VII/1951 and 7/VIII/1950 (Bechyné 1954); El Boquerón, 10/VI/1959 (Bechyné and Bechyné 1960); San Salvador, NHMW (Santisteban 2006); Volcán San Salvador USNM (Santisteban 2006). SAN VICENTE: Santa Cruz Porrillo, 14/X/1956, Berry, RA., USNM (Santisteban 2006). SANTA ANA: Hacienda Los Planes, 1800 m, Metapan Mts., 24–25/VIII/1951 (Bechyné 1954). Hacienda Los Planes, 2000 m, 30/X/1950 (Bechyné 1954); Trifinio, 12/X/1959 (Bechyné and Bechyné 1960), 12/XII/1956 and 14/VIII/1959 Berry, RA., USNM (Santisteban 2006); Volcán Santa Ana, 30/VIII/1956, Berry, RA.,USNM (Santisteban 2006); Laguna de las Ranas, 1750 m, 27/VI/1951 (Bechyné 1954); Volcan Santa Ana, W slope, 1600–1700 m, near Buenos Aires, 3/VIII/1951 (Bechyné 1954); Cerro Verde, 7/VIII/1964, Vega, J.C., Jr., TAMU (Santisteban 2006), 8/X/1956, Berry, RA, USNM (Santisteban 2006); 101.2 mi down from Cerro Verde summit, 20/VIII/1972, Hevel, GE & S, USNM (Santisteban 2006).

**Specimens examined.** AHUACHAPÁN: Apaneca, 15/VII/1959, Leg. J. Bechyné, Det. J. Bechyné, 20 spec. (RBINS).

**141. *Monomacradixira* Bechyné & Bechyné, 1963**

**Published records.** AHUACHAPÁN: Apaneca, 14–17/VII/1959 (Bechyné and Bechyné 1963). SANTA ANA: Cerro Verde, 16/V/1960 (Bechyné and Bechyné 1963).

**142. *Monomacraguazapa* Bechyné & Bechyné, 1963**

**Published records.** SAN SALVADOR: Guazapa, 10/IX/1959 (Bechyné and Bechyné 1963).

**143. *Monomacravariabilis* (Jacoby, 1884)**

**Published records.** AHUACHAPÁN: Apaneca, 14–17/VII/1959 (Bechyné and Bechyné 1963). CHALATENANGO: La Palma, 7–9/VII/1959 (Bechyné and Bechyné 1963). LA LIBERTAD: Comasagua, 1/VII/1959 (Bechyné and Bechyné 1963). SAN SALVADOR: Capital, 8/VI/1959, 13/VI/1959, 19/VI/1959, 20/VII/1959, 1/I/1960 (Bechyné and Bechyné 1963).

**144. *Monomacraviolacea* (Jacoby, 1884)**

**Published records.** SAN SALVADOR: Capital, 6/VII/1959 (Bechyné and Bechyné 1963).

**145. *Neothonaquatuordecima* Bechyné & Bechyné, 1960**

**Published records.** CHALATENANGO: La Palma, 7/VII/1959 (Bechyné and Bechyné 1960).

**146. *Neothonaquindecima* Bechyné & Bechyné, 1960**

**Published records.** LA PAZ: Volcan San Vicente, Finca La Paz, 1–10/VIII/1959 (Bechyné and Bechyné 1960). SAN SALVADOR: 8/VI/1959, 6/VII/1959, 12–13/VII/1959, 24/VII/1959, 3/X/1959 (Bechyné and Bechyné 1960). SANTA ANA: Volcan San Diego, 24/VI/1959 (Bechyné and Bechyné 1960).

**147. *Neothonasedecima* Bechyné & Bechyné, 1960**

**Published records.** MORAZÁN: Perquín, 22/IX/1959 (Bechyné and Bechyné 1960). LA PAZ: Volcan San Vicente, Finca La Paz, 1–10/VIII/1959 (Bechyné and Bechyné 1960).

**148. *Neothonatredecima* Bechyné & Bechyné, 1960**

**Published records.** CHALATENANGO: La Palma, 7/VII/1959 (Bechyné and Bechyné 1960).

**149. *Omophoitaaffinis* (Jacoby, 1880)**

**Published records.** AHUACHAPÁN: Apaneca, 14–17/VII/1959 (Bechyné and Bechyné 1960).

**150. *Omophoitaclerica* (Erichson, 1848)**

**Published records.** Bechyné and Bechyné (1961) mention the occurrence of *O.clerica* in El Salvador, but do not give specific records.

**151. *Omophoitapunctulata* (Bechyné & Bechyné, 1963)**

**Published records.** AHUACHAPÁN: Apaneca, 27/I/1960 (Bechyné and Bechyné 1963). CHALATENANGO: La Palma, 7–8/VII/1959 (Bechyné and Bechyné 1963).SAN SALVADOR: Capital, 1/I/1960 (Bechyné and Bechyné 1963); Guazapa, 10/IX/1959 (Bechyné and Bechyné 1963). SANTA ANA: Volcan San Diego, 22–29/VI/1959 (Bechyné and Bechyné 1963).

**152. *Omophoitaquadrinotatacostaricensis* (Bechyné, 1955)**

**Published records.** AHUACHAPÁN: Apaneca, 14–17/VII/1959 (Bechyné and Bechyné 1963). LA LIBERTAD: Comasagua, 1/VII/1959 (Bechyné and Bechyné 1963).

**153. *Omophoitasimulans* (Jacoby, 1892)**

**Published records.** AHUACHAPÁN: Apaneca, 14–17/VII/1959 (Bechyné and Bechyné 1963). CHALATENANGO: La Palma, 8/VII/1959 (Bechyné and Bechyné 1963). LA PAZ: Volcan San Vicente, Finca La Paz, 1–10/VIII/1959 (Bechyné and Bechyné 1963). SAN SALVADOR: Capital, 20/VI/1959 (Bechyné and Bechyné 1963). SANTA ANA: Volcan San Diego, 22/VI/1959 (Bechyné and Bechyné 1963).

**154. *Oreinoderaaptera* Bechyné & Bechyné, 1963 (Fig. 5b)**

**Published records.** SANTA ANA: Trifinio, 11–13/X/1959 and 6/III/1960 (Bechyné and Bechyné 1963).

**Specimens examined.** SANTA ANA: Trifinio, 6/III/1960, Leg. J. Bechyné, Det. J. Bechyné, Paratype, 20 spec. (RBINS).

**155. *Phrynocephaaustralis* Gilbert, 2011**

**Published records.** CUSCATLÁN: Rosario (El Rosario), 15/VI/1953, No. 444-273, Col. M.S.V. (Gilbert 2011).

**156. *Phrynocephalaevicollis* Jacoby, 1884 (Fig. 5c)**

**Published records.** SAN SALVADOR: El Boquerón, 25/V/1960 (Bechyné and Bechyné 1960). SANTA ANA: Volcan San Diego, 22–24/VI/1959 (Bechyné and Bechyné 1960).

**Specimens examined.** SAN SALVADOR: El Boquerón, 25/V/1960, Leg. J. Bechyné, Det. J. Bechyné, 52 spec. (RIBNS).

**157. *Physimerusfemoralis* Jacoby, 1886 (Fig. 5d)**

**Published records.** SAN SALVADOR: Capital, 9/VI/1959, 12–13/VI/1959, 18/VI/1959, 6/VII/1959, 20/VII/1959 (Bechyné and Bechyné 1963); El Boquerón, 25/V/1960 (Bechyné and Bechyné 1963); Guazapa, 10/IX/1959 (Bechyné and Bechyné 1963).

**Specimens examined.** SAN SALVADOR: El Boquerón, 25/V/1960, Leg. J. Bechyné, Det. J. Bechyné, 28 spec. (RBINS), 23/V/1960, Leg. J. Bechyné, Det. J. Bechyné, 17 spec. (RBINS).

**Remarks.** The examined spec. from the RBINS are labeled as *Thrasygoeusfemoralis*, which has been synonymized with *Physimerus*; see Furth and Savini (1996).

**158. *Platiprosopusacutangulus* (Chevrolat, 1834) (Fig. 5e)**

**Published records.** LA LIBERTAD: Comasagua, 1/VII/1959 (Bechyné and Bechyné 1963); Santa Tecla (Berry 1957). LA UNIÓN: Cutuco, 2–4/VI/1959 (Bechyné and Bechyné 1963). SAN SALVADOR: Capital, 30/VI/1959, 28/VII/1959, 19/VI/1960 (Bechyné and Bechyné 1963); Lago Hopango, 24/VII/1959 (Bechyné and Bechyné 1963); Guazapa, 11/V/1960 (Bechyné and Bechyné 1963). SANTA ANA: Volcan San Diego, 22–29/VI/1959 (Bechyné and Bechyné 1963).

**Specimens exami**ned. LA UNIÓN: Cutuco, 4/VI/1959, Leg. J. Bechyné, Det. J. Bechyné, 15 spec. (RBINS). UNKNOWN PROVINCE: Cartagena, 19/V/1959, Leg. J. Bechyné, Det. J. Bechyné, 17 spec. (RBINS).

**Remarks.** The spec. in the RBINS are labeled as *Phyllotrupesacutangulus*, which has been synonymized with *Platiprosopus*; see Furth and Savini (1996).

**159. *Plectotetranigripes* Jacoby, 1884**

**Published records.** SAN SALVADOR: El Boquerón, 10/VI/1959 (Bechyné and Bechyné 1963).

**160. *Plectrotetrasurquia* Bechyné & Bechyné, 1963 (Fig. 5f)**

**Published records.** AHUACHAPÁN: Apaneca, 14–17/VII/1959 (Bechyné and Bechyné 1963). CHALATENANGO: La Palma, 9/VII/1959 (Bechyné and Bechyné 1963). SANTA ANA: Cerro Verde, 16/V/1960 (Bechyné and Bechyné 1963).

**Specimens examined.** SANTA ANA: Cerro Verde, 16/V/1960, Leg. J. Bechyné, Det. J. Bechyné, Paratype, 58 spec. (RBINS).

**161. *Podalticaharrietta* Bechyné & Bechyné, 1963**

**Published records.** LA LIBERTAD: Hacienda Argentina, 17/VI/1959 (Bechyné and Bechyné 1963).

**162. *Resistencianacardiophora* Bechyné & Bechyné, 1960**

**Published records.** SAN SALVADOR: El Boquerón, 10/VI/1959 (Bechyné and Bechyné 1960).

**163. *Resistencianaornata* (Jacoby, 1884)**

**Published records.** CHALATENANGO: La Palma, 7/VII/1959 (Bechyné and Bechyné 1963). SAN SALVADOR: Capital, 6/VII/1959 (Bechyné and Bechyné 1963). SANTA ANA: Volcan San Diego, 23/VI/1959 (Bechyné and Bechyné 1963).

**164. *Styrepitrixboqueronica* Bechyné & Bechyné, 1963**

**Published records.** AHUACHAPÁN: Apaneca, 14–17/VII/1959 (Bechyné and Bechyné 1963). SAN SALVADOR: El Boquerón, 10/VI/1959 (Bechyné and Bechyné 1963). SANTA ANA: Volcan San Diego, 29/VI/1959 (Bechyné and Bechyné 1963).

**165. *Syphreaarevaloi* Bechyné & Bechyné, 1960**

**Published records.** AHUACHAPÁN: Apaneca, 14–17/VII/1959 (Bechyné and Bechyné 1960). SAN SALVADOR: 8/VI/1959, 20/VI/1959, 12/VII/1959, 24/VII/1959, 20/VIII/1959 (Bechyné and Bechyné 1960). SANTA ANA: Volcan San Diego, 29/VI/1959 (Bechyné and Bechyné 1960).

**166. *Syphreaarhenia* Bechyné & Bechyné, 1960**

**Published records.** LA PAZ: Volcan San Vicente, Finca La Paz, 1–10/VIII/1959 (Bechyné and Bechyné 1960).

**167. *Syphreabalnearia* Bechyné & Bechyné, 1960 (Fig. 5g)**

**Published records.** LA LIBERTAD: Los Chorros, 29/VI/1959 (Bechyné and Bechyné 1960); San Andrés, 15/VI/1959 (Bechyné and Bechyné 1960); Hacienda Chanmico, 20/VI/1960 (Bechyné and Bechyné 1960). LA PAZ: Volcan San Vicente, Finca La Paz, 1–10/VIII/1959 (Bechyné and Bechyné 1960). SAN SALVADOR: El Boquerón, 16/VI/1959 and 20/VIII/1959 (Bechyné and Bechyné 1960).

**Specimens examined.** LA LIBERTAD: Hacienda Chanmico, 20/VI/1960, Leg. J. Bechyné, Det. J. Bechyné, Paratype, 3 spec. (RBINS).

**168. *Syphreachrysoderma* Bechyné & Bechyné, 1960**

**Published records.** SANTA ANA: Volcan San Diego, 22–29/VI/1959 (Bechyné and Bechyné 1960).

**169. *Syphrea frígida* Bechyné & Bechyné, 1960**

**Published records.** CHALATENANGO: La Palma, 7/VII/1959 (Bechyné and Bechyné 1960).

**170. *Syphreafulvitarsis* Jacoby, 1891**

**Published records.** CHALATENANGO: La Palma, 9/VII/1959 (Bechyné and Bechyné 1960). LA PAZ: Volcan San Vicente, Finca La Paz, 1–10/VII/1959 (Bechyné and Bechyné 1960). SAN SALVADOR: 11/VI/1959, 13/VI/1959, 19/VI/1959, 30/VI/1959, 6/VII/1959, 20/VII/1959 (Bechyné and Bechyné 1960). LA LIBERTAD: Comasagua, 1/VII/1959 and 3/VII/1959 (Bechyné and Bechyné 1960).

**171. *Syphreaidiolepis* Bechyné & Bechyné, 1960**

**Published records.** LA LIBERTAD: Comasagua, 1/VII/1959 (Bechyné and Bechyné 1960). MORAZÁN: Perquín, 22/IX/1959 (Bechyné and Bechyné 1960).

**172. *Syphreapalaciosi* Bechyné & Bechyné, 1960**

**Published records.** CHALATENANGO: La Palma, 9/VII/1959 (Bechyné and Bechyné 1960).

**173. *Syphreapalomita* Bechyné & Bechyné, 1960**

**Published records.** SANTA ANA: Volcan San Diego, 23/VI/1959 (Bechyné and Bechyné 1960).

**174. *Syphreapretiosa* Baly, 1876 (Fig. 5h)**

**Published records.** CHALATENANGO: La Palma, 9/VII/1959 (Bechyné and Bechyné 1960). LA PAZ: Volcan San Vicente, Finca La Paz, 1–10/VIII/1959 (Bechyné and Bechyné 1960). SANTA ANA: Volcan San Diego, 24/VI/1959 (Bechyné and Bechyné 1960).

**Specimens examined.** CHALATENANGO: La Palma, 9/VII/1959, Leg. J. Bechyné, Det. J. Bechyné, 9 spec. (RBINS).

**175. *Syphreaquintanillai* Bechyné & Bechyné, 1960 (Fig. 5i)**

**Published records.** LA LIBERTAD: Los Chorros, 29/VI/1959 (Bechyné and Bechyné 1960); Hacienda Chanmico, 20/VI/1960 (Bechyné and Bechyné 1960).SAN SALVADOR: Lago Ilopango, 24/VII/1959 (Bechyné and Bechyné 1960); Capital, 30/VI/1959 (Bechyné and Bechyné 1960).

**Specimens examined.** LA LIBERTAD: Hacienda Chanmico, 20/VI/1960, Leg. J. Bechyné, Det. J. Bechyné, Paratype, 15 spec. (RBINS).

**176. *Syphrearufobadia* Bechyné & Bechyné, 1960**

**Published records.** SAN SALVADOR: Capital, 13/VI/1959 and 30/VI/1959 (Bechyné and Bechyné 1960); Lago Ilopango, 24/VII/1959 (Bechyné and Bechyné 1960).

**177. *Syphreasuntia* Bechyné & Bechyné, 1960**

**Published records.** CHALATENANGO: La Palma, 7/VII/1959 (Bechyné and Bechyné 1960).

**178. *Syphreateapensis* Jacoby, 1891**

**Published records.** SAN SALVADOR: Capital, 19–20/VI/1959 (Bechyné and Bechyné 1960); Guazapa, 10/IX/1959 (Bechyné and Bechyné 1960). SANTA ANA: Volcan San Diego, 23–29/VI/1959 (Bechyné and Bechyné 1960).

**179. *Systenacandella* Bechyné & Bechyné, 1963**

**Published records.** LA PAZ: Volcan San Vicente, Finca La Paz, 1–10/VIII/1959 (Bechyné and Bechyné 1963).

**180. *Systenacostifera* Bechyné & Bechyné, 1963**

**Published records.** CHALATENANGO: La Palma, 7/VII/1959 (Bechyné and Bechyné 1963). SAN SALVADOR: Capital, 12/VI/1959 (Bechyné and Bechyné 1963).

**181. *Systenaelongatula* Csiki, 1939**

**Published records.** AHUACHAPÁN: Apaneca, 14–17/VII/1959 (Bechyné and Bechyné 1963). CHALATENANGO: La Palma, 7/VII/1959 (Bechyné and Bechyné 1963); La Palma, 9/VII/1959 (Bechyné and Bechyné 1963). LA LIBERTAD: Santa Tecla, 28/IX/1959 (Bechyné and Bechyné 1963). SAN SALVADOR: Capital, 20/VIII/1959 (Bechyné and Bechyné 1963). El Boquerón, 10/VI/1959 (Bechyné and Bechyné 1960). SANTA ANA: Trifinio, 17/X/1959 (Bechyné and Bechyné 1963).

**182. *Systenaguija* Bechyné & Bechyné, 1963**

**Published records.** CHALATENANGO: La Palma, 9/VII/1959 (Bechyné and Bechyné 1963). CUSCATLÁN: Hacienda Colima, 22/VII/1959 (Bechyné and Bechyné 1963). MORAZÁN: Perquín, 27/IX/1959 (Bechyné and Bechyné 1963). SAN SALVADOR: Cerro San Jacinto, 17/IX/1959 (Bechyné and Bechyné 1963). SANTA ANA: Volcan San Diego, 23–29/VI/1959 (Bechyné and Bechyné 1963).

**183. *Systenalepontina* Bechyné & Bechyné, 1963**

**Published records.** CHALATENANGO: La Palma, 8/VII/1959 (Bechyné and Bechyné 1963). LA LIBERTAD: Comasagua, 3/VII/1959 (Bechyné and Bechyné 1963); Los Chorros, 29/VI/1959 (Bechyné and Bechyné 1963). SAN SALVADOR: Capital, 7–8/VI/1959, 18–19/VI/1959, 30/VI/1959, 3/V/1960, 15/V/1960, 18/V/1960 (Bechyné and Bechyné 1963).

**184. *Systenamelanosterna* Bechyné & Bechyné, 1963**

**Published records.** CHALATENANGO: La Palma, 7/VII/1959 and 9/VII/1959 (Bechyné and Bechyné 1963). SAN SALVADOR: Cerro San Jacinto, 17/IX/1959 (Bechyné and Bechyné 1963).

**185. *Systenapectoralis* Clark, 1865**

**Published records.** CHALATENANGO: La Palma, 7/VII/1959 (Bechyné and Bechyné 1963).

**186. *Systenasulcatula* Bechyné & Bechyné, 1963**

**Published records.** LA LIBERTAD: Comasagua, 1/VII/1959 (Bechyné and Bechyné 1963). SAN SALVADOR: El Boquerón, 10/VI/1959 (Bechyné and Bechyné 1963).

**187. *Systenathoracica* Jacoby, 1884**

**Published records.** AHUACHAPÁN: Apaneca, 14–17/VII/1959 (Bechyné and Bechyné 1963); Los Ausoles, 26/I/1960 (Bechyné and Bechyné 1963). LA PAZ: Volcan San Vicente, Finca La Paz, 1–10/VIII/1959 (Bechyné and Bechyné 1963). MORAZÁN: Perquín, 22/IX/1959 (Bechyné and Bechyné 1963). SANTA ANA: Volcan San Diego, 24/VI/1959 (Bechyné and Bechyné 1963).

**188. *Systenavariabilis* Jacoby, 1884 (Fig. 5j)**

**Published records.** CHALATENANGO: La Palma, 7–8/VII/1959 (Bechyné and Bechyné 1963). SAN SALVADOR: Capital, 23/V/1960 (Bechyné and Bechyné 1963); El Boquerón, 10/VI/1959 (Bechyné and Bechyné 1963). SANTA ANA: Volcan San Diego, 27/VI/1959 (Bechyné and Bechyné 1963).

**Specimens examined.** SAN SALVADOR: 23/V/1960, Leg. J. Bechyné, Det. J. Bechyné, 30 spec. (RBINS).

**Remarks.**Berry (1959) also mentions this species as being present in El Salvador, but does not give specific records.

**189. *Temnocrepistrifiniensis* Bechyné & Bechyné, 1963**

**Published records.** SANTA ANA: Trifinio,11–13/V/1959 (Bechyné and Bechyné 1963).

**190. *Trichalticavariabilis* Jacoby, 1885**

**Published records.** AHUACHAPÁN: Apaneca, 14–17/VII/1959 (Bechyné and Bechyné 1963). SAN SALVADOR: Capital, 19/VI/1960 (Bechyné and Bechyné 1963).

**191. *Trifiniocolafreundi* Bechyné & Bechyné, 1963**

**Published records.** SANTA ANA: Trifinio, 10–14/X/1959 and 8–9/III/1960 (Bechyné and Bechyné 1963).

**192. *Walterianellabiarcuata* (Chevrolat, 1834)**

**Published records.** MORAZÁN: Perquín, 22/IX/1959 (Bechyné and Bechyné 1963).

**193. *Walterianellaexocosta* Bechyné & Bechyné, 1963**

**Published records.** SAN SALVADOR: Captital, 1/I/1960, 24/IV/1960, 15/V/1960, 23/V/1960 (Bechyné and Bechyné 1963); Guazapa, 10/IX/1959 (Bechyné and Bechyné 1963).

**194. *Walterianellahypocrita* (Jacoby, 1886)**

**Published records.** CUSCATLÁN: El Rosario (Berry 1957).

**195. *Walterianellainscripta* (Jacoby, 1886)**

**Remarks.**Berry (1959) mentions this species as being present in El Salvador, but does not give specific records.

**196. *Walterianellasublineata* (Jacoby, 1886)**

**Published records.** AHUACHAPÁN: Apaneca, 14–17/VII/1959 (Bechyné and Bechyné 1963). SAN SALVADOR: El Boquerón, 10/VI/1959 (Bechyné and Bechyné 1963). Usulután: Alegría, 27/II/1960 (Bechyné and Bechyné 1963).

**197. *Walterianellatenuicincta* (Jacoby, 1886)**

**Published records.** AHUACHAPÁN: Apaneca, 14–17/VII/1959, (Bechyné and Bechyné 1963). SAN SALVADOR: Capital, 8/IX/1959 (Bechyné and Bechyné 1963).

**198. *Walterianellavenustula* (Schaufuss, 1874) (Fig. 4k)**

**Published records.** LA LIBERTAD: Hacienda Argentina, 15/VI/1959 and 17/VI/1959 (Bechyné and Bechyné 1963). LA PAZ: Volcan San Vicente, Finca La Paz, 1–10/VIII/1959 (Bechyné and Bechyné 1963). SAN SALVADOR: Capital, 11/VI/1959, 19/VI/1959, 30/VI/1959, 28/VII/1959, 24/IV/1960, 29/IV/1960 (Bechyné and Bechyné 1963); Lago Ilopango, 24/VII/1959 (Bechyné and Bechyné 1963); Guazapa, 10/IX/1959 (Bechyné and Bechyné 1963). SANTA ANA: Trifinio, 14/X/1959 (Bechyné and Bechyné 1963).

**Specimens examined.** SAN SALVADOR: 24/IV/1960, Leg. J. Bechyné, Det. J. Bechyné, 63 spec. (RBINS).

#### Subfamily Galerucinae excl. Alticini

**Figure 6.**
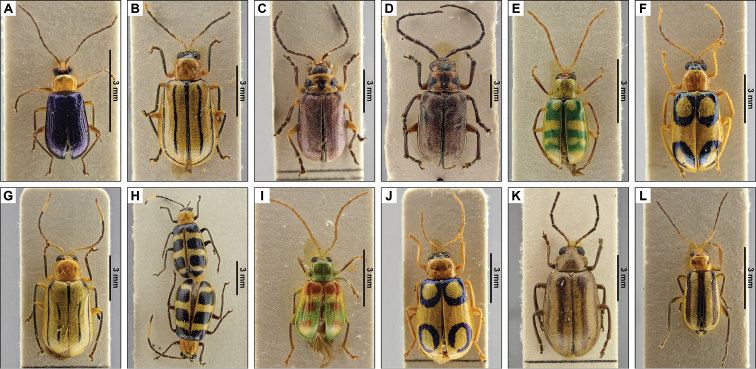
Dorsal pictures of the species of Galerucinae excluding Alticini from El Salvador currently present in the collections of the RBINS. **A***Acalymmasemicaerulea***B***Amphelasmanigrolineatum***C***Coraiaclarki***D***C.maculicollis***E***Diabroticabalteata***F***D.circulata***G***D.curvilineata***H***D.pulchra***I***D.viridula***J***Neobroticaornata***K***Pyesiadetritalaevicollis*. High resolution images can be found at http://collections.naturalsciences.be/ssh-entomology.

**1. *Acalymmasemicoerulea* (Jacoby, 1887) new record (Fig. 6A)**

**Specimens examined.** SAN SALVADOR: Cerro San Jacinto, 17/IX/1959, Leg. J. Bechyné, Det. J. Bechyné, 11 spec. (RBINS), 2/VII/1960, Leg. J. Bechyné, Det. J. Bechyné, 10 spec. (RBINS).

**2. *Acalymmatrivittata* (Mannerheim, 1843)**

**Published records.** LA LIBERTAD: Santa Tecla (Berry 1957).

**3. *Amphelasmanigrolineatum* (Jacoby, 1878) new record (Fig. 6B)**

**Specimens examined.** SAN SALVADOR: 23/V/1960, Leg. J. Bechyné, Det. J. Bechyné, 7 spec. (RBINS). SANTA ANA: Trifinio, 6/III/1960, Leg. J. Bechyné, Det. J. Bechyné, 12 spec. (RBINS).

**4. *Cerotomaatrofasciata* Jacoby, 1879**

**Remarks.**Berry (1959) mentions this species as being present in El Salvador, but does not give specific records.

**5. *Cerotomaruficornis* (Olivier, 1791)**

**Published records.** LA UNIÓN: La Unión (Jacoby 1880 – 1892).

**6. *Coraiaclarki* Jacoby, 1886 new record (Fig. 6C)**

**Specimens examined.** LA LIBERTAD: Hacienda Argentina, 18/V/1960, Leg. J. Bechyné, Det. J. Bechyné, 93 spec. (RBINS).

**7. *Coraiamaculicollis* Clark, 1865 new record (Fig. 6D)**

**Specimens examined.** LA LIBERTAD: Hacienda Argentina, 18/V/1960, Leg. J. Bechyné, Det. J. Bechyné, 4 spec., (RBINS).

**8. *Diabroticaamecameca* Krysan & Smith, 1987**

**Remarks.**Derunkov et al. (2013) list El Salvador under known distribution of *D.amecameca*, but do not give specific localities.

**9. *Diabroticabalteata* LeConte, 1865 (Fig. 6E)**

**Published records.** LA LIBERTAD: Santa Tecla (Berry 1957); San Andrés (Berry 1957). SAN SALVADOR: Aeropuerto de Ilopango (Berry 1957). SAN VICENTE: Santa Cruz Porrillo (Berry 1957).

**Specimens examined.** LA LIBERTAD: Zapotitan, 22/I/1960, Leg. J. Bechyné, Det. J. Bechyné, 11 spec. (RBINS). SAN SALVADOR: 1/I/1960, Leg. J. Bechyné, Det. J. Bechyné, 4 spec. (RBINS). UNKNOWN PROVINCE: Asino, Lago Ilopango, 24/VII/1959, Leg. J. Bechyné, Det. J. Bechyné, 8 spec. (RBINS).

**10. *Diabroticacirculata* Harold, 1875 new record (Fig. 6F)**

**Specimens examined.** SAN SALVADOR: 23/V/1960, Leg. J. Bechyné, Det. J. Bechyné, 8 spec. (RBINS). SANTA ANA: Trifinio, 6/III/1960, Leg. J. Bechyné, Det. J. Bechyné, 13 spec. (RBINS).

**11. *Diabroticacurvilineata* Jacoby, 1887 new record (Fig. 6G)**

**Specimens examined.** SAN SALVADOR: 23/V/1960, Leg. J. Bechyné, Det. J. Bechyné, 10 spec. (RBINS).

**12. *Diabroticalitterata* (Sahlberg, 1823)**

**Published records.** CUSCATLÁN: El Rosario (Berry 1957).

**13. *Diabroticaporracea* (Harold, 1875)**

**Remarks.**Derunkov et al. (2013) list El Salvador under known distribution of *D.porracea*, but do not give specific localities. Berry (1959) also mentions this species as being present in El Salvador, but does not give specific records.

**14. *Diabroticapulchra* (Sahlberg, 1823) (Fig. 6H)**

**Published records.** CUSCATLÁN: El Rosario (Berry 1957).

**Specimens examined.** USULUTÁN: Alegría, 22/II/1960, Leg. J. Bechyné, Det. J. Bechyné, 26 spec. (RBINS).

**Remarks.**Derunkov et al. (2013) list El Salvador under known distribution of *D.pulchra*, but do not give specific localities. The record listed in Berry and Salazar (1957) is listed as *D.albosignata* which is a synonym of *D.pulchra*.

**15. *Diabroticasalvadorensis* Derunkov et al., 2015**

**Published records.** CUSCATLÁN: Rosario Cuscatlan, No 444–506B, 17/III/1955, Col. M.S.V. (Derunkov et al. 2015); Rosario Cuscatlan, No 444–218A, 20/XI/1953, Col. M.S. (Derunkov et al. 2015). LA UNIÓN: No 444–766A, 14/V/1954, Col. C.A.B. (Derunkov et al. 2015). SAN SALVADOR: No 714.210, 14/III/1957, Col. PAB, det. R.F. Smith 1963, *Diabrotica* n. sp. near *D.pulchra* (Sahlberg) (Derunkov et al. 2015). SANTA ANA: 6.0 km W. Hwy CA1 above Lago de Coatepeque, 853 mts., 1/VI/1973 (Derunkov et al. 2015). USULUTÁN: 4 miN Santiago de Maria, 29/VIII/1972, G.F.and S. Hevel. (Derunkov et al. 2015).

**16. *Diabrotica spec.iosa spec.iosa* (Germar, 1824)**

**Remarks.**Derunkov et al. (2013) list El Salvador under known distribution of *D.pulchra*, but do not give specific localities.

**17. *Diabroticaviridula* (Fabricius, 1801) (Fig. 6I)**

**Published records.** USULUTÁN: Berlín (Bechyné and Bechyné 1962).

**Specimens examined.** SAN SALVADOR: Cerro San Jacinto, 17/IX/1959, Leg. J. Bechyné, Det. J. Bechyné, 5 spec. (RBINS). USULUTÁN: Alegría, 22/II/1960, Leg. J. Bechyné, Det. J. Bechyné, 23 spec. (RBINS).

**Remarks.**Derunkov et al. (2013) also lists El Salvador under known distribution of *D.viridula*, but does not give specific localities. Bechyné and Bechyné (1969a) state that *D.viridula* is common in San Salvador. Berry (1959) also mentions this species as being present in El Salvador, but does not give specific records as well.

**18. *Exoradetrita* (Fabricius, 1801)**

**Published records.** CUSCATLÁN: Finca El Carmen, 1300 m, Volcan San Vicente, 11–16/VI/1951 (Bechyné 1954).

**19. *Exoraencaustica* Harold, 1875**

**Published records.** LA LIBERTAD: Santa Tecla (Berry 1957).

**Remarks.**Bechyné and Bechyné (1962) list El Salvador under species distribution, but do not give specific localities.

**20. *Exoraobsoleta* (Fabricius, 1801)**

**Published records.** SAN SALVADOR: 22/VII/1951 (Bechyné 1954).

**Remarks.**Bechyné and Bechyné (1962) also list El Salvador under species distribution, but do not give specific localities. This thus might be a reference to the locality listed above.

**21. *Gynandrobroticanigrofasciata* (Jacoby, 1878)**

**Published records.** CUSCATLÁN: El Rosario (Berry 1957). LA LIBERTAD: Los Chorros (Berry 1957).

**22. *Gynandrobroticavariabilis* (Jacoby, 1887)**

**Published records.** CUSCATLÁN: El Rosario (Berry 1957).

**23. *Monocestaducalis* Clark, 1865**

**Published records.** SAN SALVADOR: 8/VII/1951 (Bechyné 1954).

**Remarks.**Berry (1959) also mentions this species as being present in El Salvador, but does not give specific records.

**24. *Monocestajansoni* Jacoby, 1886**

**Published records.** LA LIBERTAD: Santa Tecla (Berry 1957).

**25. *Monoleptabipartita* Jacoby, 1888**

**Published records.** CUSCATLÁN: El Rosario (Berry 1957).

**26. *Neobroticaornata* Jacoby, 1887 new record (Fig. 6J)**

**Specimens examined.** SAN SALVADOR: 23/V/1960, Leg. J. Bechyné, Det. J. Bechyné, 5 spec. (RBINS).

**27. *Pyesiadetritalaevicollis* (Jacoby, 1887) (Fig. 6K)**

**Specimens examined.** SAN SALVADOR: 1/I/1960, Leg. J. Bechyné, Det. J. Bechyné, 1 spec. (RBINS), 1/I/1960, Leg. J. Bechyné, Det. J. Bechyné, 25 spec. (RBINS).

**Remarks.**Bechyné and Bechyné (1962) list El Salvador under species distribution, but do not give specific localities.

**28. *Trichobroticasexplagiata* (Jacoby, 1878) new record**

**Specimens examined.** SAN SALVADOR: 27/II/1959, Leg. J. Bechyné, Det. J. Bechyné, 20 spec. (RBINS).

#### Subfamily Lamprosomatinae

**1. *Lamprosomasplendidum* Lacordaire, 1848**

**Remarks.**Berry (1959) mentions this species as being present in El Salvador, but does not give specific records.

## Discussion

The digitization of the Bechyné collection in the RBINS revealed that there were a total of 2797 individual specimens sorted into 89 species. Among these were ten apparent nomina nuda which could not be found in any publication from J. Bechyné: *Antitypona* sp. (Manuscript species – Eumolpinae), *Brachypnoea* sp. *1* (Manuscript species – Eumolpinae), *Brachypnoea* sp. *2* (Manuscript species – Eumolpinae), *Hylax* sp. (Manuscript species – Eumolpinae), *Percolaspis* sp. *1* (Manuscript species – Eumolpinae), *Percolaspis* sp. *2* (Manuscript species – Eumolpinae), *Phanaeta* sp. (Manuscript species – Eumolpinae), *Chaetocnema* sp. (Manuscript species – Galerucinae, Alticini), *Phyllotreta* sp. (Manuscript species – Galerucinae, Alticini) and *Walterianella* sp. (Manuscript species – Galerucinae, Alticini). These are species which Bechyné either forgot to formerly describe, did not have time to describe, or the validity of which he questioned. Regarding the former list, the authors believe that *Walterianella* sp. (Manuscript species) could just be a variation of *W.venustula* (Schaufuss) which it closely resembles, but this should be confirmed by comparison with the type material and especially the structure of the genital structures. These and all of the other “paratypes” of the *nomina nuda* should be examined and revised in the future by experts.

The study of relevant literature led to a checklist of 385 species known from El Salvador. A total of 43 species from these 309 were also present in the collections in the RBINS. Material from the Bechyné collection added a further 33 species (excluding the ten *nomina nuda*) to the literature-based checklist of chrysomelids. This leads to a preliminary checklist of a total of 420 species of Chrysomelidae currently known for El Salvador (see table 1 for a full overview). Incorporated were also records from Berry and Salazar (1957) and Berry (1959), references which were frequently overlooked in the past.

Surprisingly few records of the subfamilies Criocerinae, Lamprosomatinae, and Cryptocephalinae could be found, despite their high prevalence in Central America. This is most likely due to the fact that most chrysomelid research in El Salvador has been done by J. and B. Bechyné, who focused mostly on Eumolpinae and Galerucinae including Alticini (of which respectively 16, 9, and 2 species could be newly added to the El Salvador checklist by Bechyné’s collection in the RBINS). The latter are relatively well represented in comparison with the currently known number of species from neighbouring countries (see Furth and Savini (1996)), mainly because of the extensive work of the Bechynés.

We noted a strong bias towards collection efforts in the departments San Salvador (182 species), La Libertad (105 species) and Santa Ana (114 species), and to a lesser extent in the districts Ahuachapán (54 species), La Paz (50 species) and Chalatenango (46 species). No records from San Miguel or Cabañas could be found. Future surveys in the country should thus also be focussed on the two latter departments.

## Conclusions

Our study reveals a preliminary total of 420 species of Chrysomelidae known to El Salvador. However, this number should be approached with caution, since the taxonomy of some subfamilies is not yet fully clear (e.g., Eumolpinae), some subfamilies seem to lack sampling effort in the country (e.g., Cryptocephalinae, Criocerinae and Lamprosomatinae), and in general there has been little study on the fauna of El Salvador, possibly due to its political instability and safety issues for field research (Bourgois 2001). Nonetheless, we believe that this checklist, although almost certainly incomplete, will serve as a baseline for further study in the area.

## Supplementary materials

Supplementary material 1 Table S1. Species and genera count per subfamily for El Salvador and each of El Salvador’s departments Data type: species data Explanation note: Species given for department “Unknown” are species counts for which there were country records only. This dataset is made available under the Open Database License (http://opendatacommons.org/licenses/odbl/1.0/). The Open Database License (ODbL) is a license agreement intended to allow users to freely share, modify, and use this Dataset while maintaining this same freedom for others, provided that the original source and author(s) are credited.Martijn Van Roie, Frederik De Wint, Ayse Güngor, Charlotte Huyghe, Wouter Dekoninck, Lukáš Sekerka
