# Supplementary material for: An annotated checklist of the leaf beetles (Coleoptera, Chrysomelidae) from El Salvador, with additions from the Bechyné collection in the Royal Belgian Institute of Natural Sciences
Source: Zookeys. 2019 Jun 17;856:137–96. doi: 10.3897/zookeys.856.32017 (PMC6591217; doi:10.3897/zookeys.856.32017)
Supplement: Supplementary material 1 [file zookeys-856-137-s001.docx]

**Supplementary Table 1:** Species and genera count per subfamily for El Salvador and each of El Salvador’s departments. Species given for department “Unknown” are species counts for which there were country records only.

| **Locality** | **Total** | | **Bruchinae** | | **Cassidinae** | | **Chrysomelinae** | | **Cryptocephalinae** | | **Eumolpinae** | | **Galerucinae - Alticini** | | **Galerucinae ss.** | | **Lamprosomatinae** | |
| --- | --- | --- | --- | --- | --- | --- | --- | --- | --- | --- | --- | --- | --- | --- | --- | --- | --- | --- |
|  | **Species** | **Genera** | **Species** | **Genera** | **Species** | **Genera** | **Species** | **Genera** | **Species** | **Genera** | **Species** | **Genera** | **Species** | **Genera** | **Species** | **Genera** | **Species** | **Genera** |
| **El Salvador** | **420** | **132** | **63** | **16** | **74** | **30** | **19** | **5** | **8** | **6** | **29** | **17** | **198** | **45** | **28** | **12** | **1** | **1** |
| Ahuachapán | 54 | 30 | - | - | 4 | 4 | 2 | 1 | - | - | - | - | 48 | 25 | - | - | - | - |
| Cabañas | - | - | - | - | - | - | - | - | - | - | - | - | - | - | - | - | - | - |
| Chalatenango | 46 | 24 | 1 | 1 | 2 | 2 | - | - | - | - | - | - | 43 | 21 | - | - | - | - |
| Cuscutlán | 40 | 28 | 1 | 1 | 9 | 8 | 4 | 3 | 3 | 3 | 4 | 3 | 12 | 6 | 7 | 4 | - | - |
| La Libertad | 105 | 65 | 11 | 8 | 33 | 20 | 6 | 3 | 2 | 2 | 3 | 3 | 43 | 23 | 7 | 6 | - | - |
| La Paz | 50 | 25 | 1 | 1 | 8 | 7 | - | - | - | - | - | - | 41 | 17 | - | - | - | - |
| La Unión | 25 | 22 | 13 | 10 | 4 | 4 | 1 | 1 | 1 | 1 | 1 | 1 | 3 | 3 | 2 | 2 | - | - |
| Morazán | 29 | 17 | - | - | 2 | 2 | 1 | 1 | - | - | - | - | 26 | 14 | - | - | - | - |
| San Miguel | - | - | - | - | - | - | - | - | - | - | - | - | - | - | - | - | - | - |
| San Salvador | 182 | 86 | 15 | 10 | 25 | 18 | 6 | 2 | 6 | 2 | 15 | 10 | 103 | 36 | 12 | 8 | - | - |
| San Vicente | 14 | 13 | 2 | 2 | 8 | 8 | - | - | - | - | - | - | 3 | 2 | 1 | 1 | - | - |
| Santa Ana | 114 | 59 | 5 | 5 | 17 | 12 | 5 | 2 | 5 | 2 | 3 | 1 | 76 | 35 | 3 | 2 | - | - |
| Sonsonate | 9 | 7 | - | - | 7 | 5 | - | - | - | - | 1 | 1 | 1 | 1 | - | - | - | - |
| Usulután | 10 | 8 | 1 | 1 | 4 | 4 | - | - | - | - | - | - | 2 | 2 | 3 | 1 | - | - |
| *Unknown* | 64 | 33 | 28 | 5 | 15 | 10 | 1 | 1 | 3 | 2 | 7 | 7 | 5 | 5 | 4 | 2 | 1 | 1 |
